# Metabolomic fingerprinting of porcine lung tissue during pre-clinical prolonged *ex vivo* lung perfusion using *in vivo* SPME coupled with LC-HRMS

**DOI:** 10.1016/j.jpha.2022.06.002

**Published:** 2022-06-08

**Authors:** Nikita Looby, Anna Roszkowska, Aadil Ali, Barbara Bojko, Marcelo Cypel, Janusz Pawliszyn

**Affiliations:** a Department of Chemistry, University of Waterloo, Waterloo, N2L 3G1, Canada; b Department of Pharmaceutical Chemistry, Medical University of Gdansk, 80-416, Gdansk, Poland; c Division of Thoracic Surgery, University Health Network, Toronto, M5G 2C4, Canada; d Department of Pharmacodynamics and Molecular Pharmacology, Faculty of Pharmacy, Collegium Medicum in Bydgoszcz, Nicolaus Copernicus University in Torun, 85-089, Bydgoszcz, Poland

**Keywords:** Normothermic ex vivo lung perfusion, Solid phase microextraction, Lung metabolomics

## Abstract

Normothermic ex vivo lung perfusion (NEVLP) has emerged as a modernized organ preservation technique that allows for detailed assessment of donor lung function prior to transplantation. The main goal of this study was to identify potential biomarkers of lung function and/or injury during a prolonged (19 h) NEVLP procedure using in vivo solid-phase microextraction (SPME) technology followed by liquid chromatography-high resolution mass spectrometry (LC-HRMS). The use of minimally invasive in vivo SPME fibers for repeated sampling of biological tissue permits the monitoring and evaluation of biochemical changes and alterations in the metabolomic profile of the lung. These in vivo SPME fibers were directly introduced into the lung and were also used to extract metabolites (on-site SPME) from fresh perfusate samples collected alongside lung samplings. A subsequent goal of the study was to assess the feasibility of SPME as an in vivo method in metabolomics studies, in comparison to the traditional in-lab metabolomics workflow. Several upregulated biochemical pathways involved in pro- and anti-inflammatory responses, as well as lipid metabolism, were observed during extended lung perfusion, especially between the 11th and 12th hours of the procedure, in both lung and perfusate samples. However, several unstable and/or short-lived metabolites, such as neuroprostanes, have been extracted from lung tissue in vivo using SPME fibers. On-site monitoring of the metabolomic profiles of both lung tissues through in vivo SPME and perfusate samples on site throughout the prolonged NEVLP procedure can be effectively performed using in vivo SPME technology.

## Highlights

• In vivo SPME monitors metabolic changes in porcine lung during 19-h NEVLP.
• On-site SPME for perfusate sampling monitors metabolite composition during NEVLP.
• SPME-LC-HRMS permits identification of potential metabolic markers of lung function.
• Stored perfusate provides less relevant metabolome information compared to on-site perfusate samples.
• In vivo SPME of the lung provides more metabolomic information than perfusate sampling.

## Graphical abstract

**Figure:**
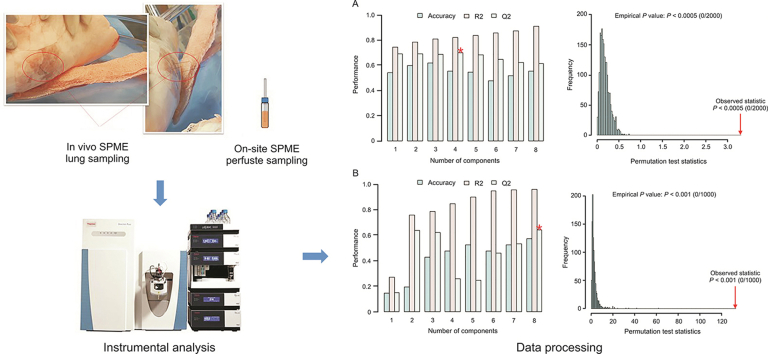

## 1 Introduction

Lung transplantation remains the single most effective personalized therapy for treating patients with end-stage pulmonary diseases such as pulmonary fibrosis, chronic obstructive pulmonary disease (COPD), emphysema, and cystic fibrosis [1,2]. However, the number of patients awaiting transplantation far exceeds the pool of available donor organs. This shortage of donor organs is exacerbated by the fact that only 20% meet the strict criteria for transplantation because of the various complications that can occur during donation after brain death and donation after cardiac death of donors, including gastric aspiration, bacterial infection, and longer warm ischemic times. Furthermore, 20% of transplanted lungs experience acute lung injury and/or primary graft dysfunction [2].

Normothermic ex vivo lung perfusion (NEVLP) is a modern organ preservation technique wherein perfusion fluid is circulated throughout the isolated lung at physiological temperature through a specialized circuit set-up. This method improves the function of lungs that are declined for transplantation by enabling innate protective metabolic processes that occur under normothermic conditions [3,4]. Additionally, NEVLP allows specific targeted therapies to be administered within the circuit through perfusion fluid to treat the above-mentioned complications or other donor-borne illnesses prior to transplantation [5]. Moreover, NEVLP allows for more detailed assessments of organ function prior to transplantation [3,4,6]. These advantages have been instrumental in increasing the donor pool, thereby decreasing patient waiting-list morbidity and mortality. Several “-omics” studies, including genomics, transcriptomics, epigenomics, and proteomics [7,8], have explored the pathophysiology of these end-stage lung diseases, specifically COPD. Other less-known studies, particularly in metabolomics, have been conducted to assess the effectiveness of the NEVLP approach itself [1]. Metabolomics is an emerging field in systems biology that can assess and elucidate the biochemical status of an organism through the analysis of all small endogenous molecules (metabolites) in that system at a pre-defined period [9]. Because changes in small metabolites are a more rapid indicator of an immediate systemic response, the use of metabolomics may potentially be more advantageous than genomics or proteomics in determining markers of improved lung function or potential graft dysfunction that may emerge over the course of NEVLP.

Previous research has shown that the evaluation of perfusate (Steen™ solution) collected periodically during the NEVLP procedure can provide biomarkers for lung function [1]. Although perfusate analysis has been shown to be useful, it is possible to obtain more information through direct tissue analysis. However, tissue analysis may be impractical because of the complexities associated with organ heterogeneity, the need for various laborious tissue homogenization techniques related to sample preparation, and the regular collection of biopsies, usually through invasive procedures. Therefore, this approach is often avoided. To overcome the limitations of typical tissue analysis workflows, in vivo solid-phase microextraction (SPME) is proposed here as a non-exhaustive, minimally invasive alternative sample preparation technique for metabolomic analyses of lung tissue during NEVLP. The simplicity and small size of the device allows for repeated insertions into the tissue over the course of the procedure, thereby eliminating the need for multiple tissue biopsies. The extraction and isolation of small molecules using SPME occurs through interaction with the polymeric extraction phase of the SPME device and the partitioning of these metabolites from the sample tissue or fluid into the extraction phase because of their affinity to the extraction phase.

The use of SPME has previously been reported in various bioanalytical and clinical applications, including tissue sampling and tissue metabolomics applications [[10], [11], [12]]. These processes include in vivo sampling and quantitation of pharmaceuticals in live fish muscle, in vivo metabolomic fingerprinting of brain tissue in living and moving rats subjected to deep brain stimulation, and in vivo therapeutic quantitation and evaluation of doxorubicin during in vivo chemo-perfusion of late-stage lung cancer patients [[13], [14], [15]]. In addition, prior research has investigated the effectiveness of various coatings for non-specific global extraction of metabolites, with findings showing that hydrophilic-lipophilic balanced (HLB) coatings can provide excellent recovery for a wide range of analytes (more polar and non-polar molecules) [16]. Thus, SPME is suitable for metabolomics, as the biocompatible coating and extraction phase type of the device allows for the exclusive extraction of small molecules, including hydrophobic and hydrophilic compounds. Various classes of small molecules, including lipids, fatty acids, amino acids, sugars, and other endogenous or exogenous molecules, can be directly extracted from live complex biological matrices, such as lung tissue, without significantly interfering with the biochemical transformations of the system under study or adversely affecting the living system itself or the sampling device [17].

In the present study, HLB-coated SPME fibers were used to sample porcine lung tissue in vivo during a prolonged (19 h) NEVLP procedure to identify potential metabolic markers of lung function, primary graft dysfunction, and/or general organ stress. Simultaneously, the perfusate was also sampled extensively on site and after storage at −80 °C using two types of SPME devices: SPME HLB fibers and HLB thin-film microextraction (TFME). This multi-faceted sampling approach aimed to provide insights into whether either lung tissue or perfusate samples are sufficient on their own to provide pertinent metabolic information, or whether it is more advantageous to employ a complementary approach that uses both types of samples simultaneously. This study also explored whether using both types of SPME devices in conjunction during on-site sample collection may be optimal for obtaining maximum information, for example, using SPME fibers for in vivo metabolomics and TFME for on-site perfusate analysis.

## 2 Materials and methods

### 2.1 Chemicals and materials

The nitinol wires used as SPME fiber supports were purchased from Confluent Inc. (Palo Alto, CA, USA), whereas the stainless-steel combs used for TFME were purchased from PAS Technologies (Magdala, Germany). Oasis HLB 5 μm particles were provided by Waters Corporation (Milford, MA, USA), and 45–60 μm HLB particles were obtained from SPE cartridges purchased from Millipore Sigma (Burlington, MA, USA). LC-MS-grade acetonitrile, methanol, and water were purchased from Millipore Sigma, as were formic acid, acetic acid, and other chemicals, including polyacrylonitrile, *N*,*N*-dimethylformamide, and hydrochloric acid. A flask-type sprayer was also purchased from Millipore Sigma.

### 2.2 Instrumental analysis

The extracts obtained from the lung and perfusate samples were analyzed using LC coupled with high resolution mass spectrometry (HRMS). Chromatographic separation was achieved on a Discovery HS F5-3 (PFP) column (10 cm × 2.1 mm, 5 μm particle size), which was protected by a guard column (2 cm × 2.1 mm, 3 μm particle size; Millipore Sigma, Darmstadt, Germany). A 40-min gradient elution with a Thermo Fisher Scientific accelerator binary pump and autosampler (Waltham, MA, USA) was used. Mobile phase A consisted of water and mobile phase B consisted of acetonitrile. For positive and negative mode polarity, 0.1% formic acid or 1 mM acetic acid was added. A Thermo Fisher Scientific Exactive Mass Spectrometer equipped with an Ion Max heated electrospray ionization source was used to perform acquisition in high-resolution (50,000) mode, with a balanced automatic gain control, an injection time of 100 ms, and a scan range of 100–1000 *m*/*z*.

Pooled quality control (QC) was prepared for each set of samples (fibers vs. TFME) by pooling 10 μL of each sample in a single vial. These pooled QC samples were injected into every 10th sample to ensure instrumental stability during the acquisition process. Samples were run randomly for both sets of data to avoid confounding the resulting data with any instrumental variations.

### 2.3 Experimental design

**Fig. 1:**
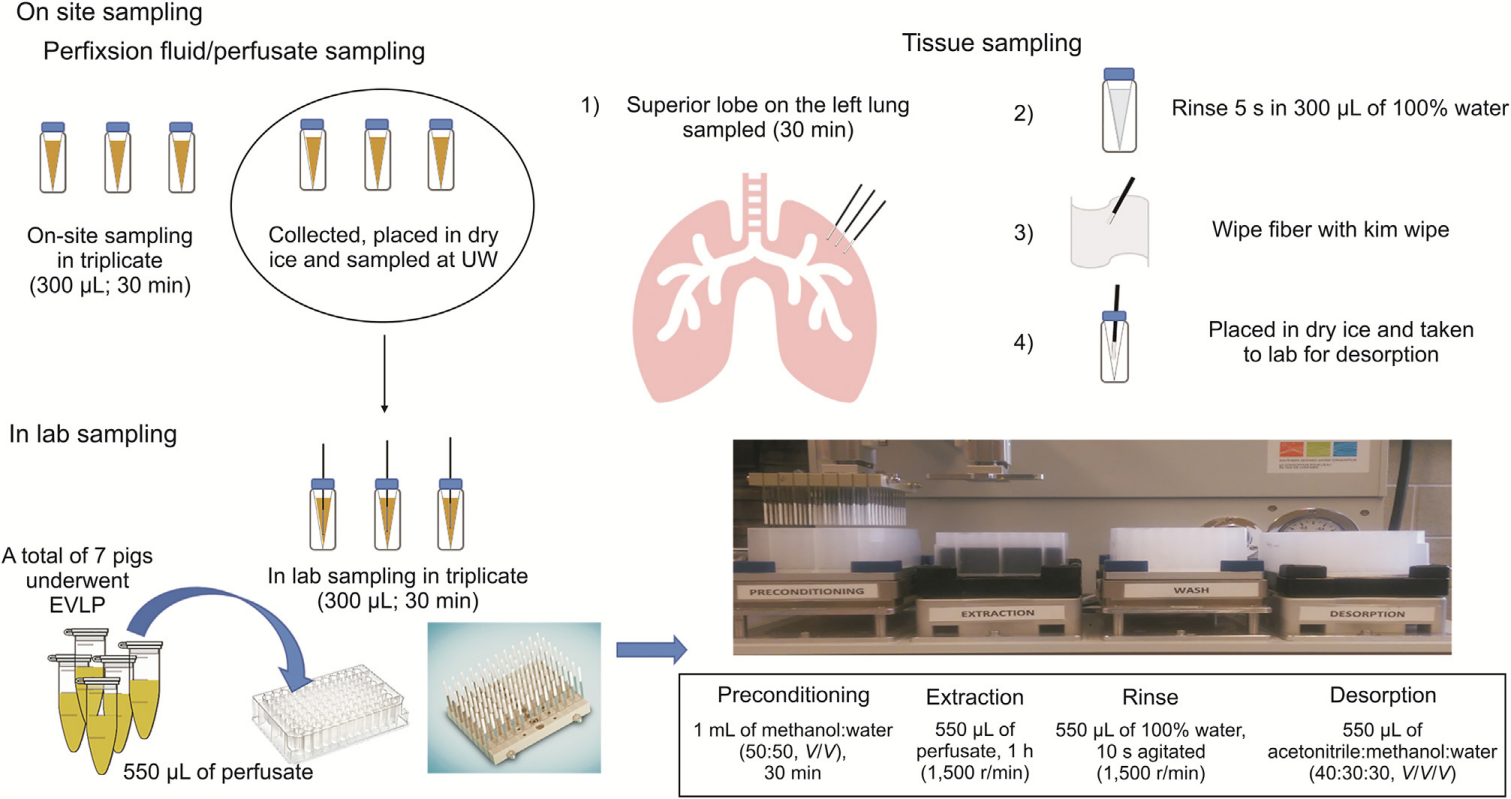
Graphical representation of the experimental design of solid-phase microextraction (SPME) sampling protocol. UW: University of Waterloo; EVLP: ex vivo lung perfusion.

The main objective of this study was to evaluate the potential of SPME technology as an in vivo and on-site extraction method for monitoring small endogenous molecules (metabolites) in lung tissue and perfusate samples during prolonged NEVLP in an in vivo pig model. First, the biochemical profile alterations in both lung and perfusate samples over the course of the NEVLP procedure were evaluated separately. Then, a compare/contrast of the data-sets was performed to determine whether a complementary approach sampling both types of matrices (lung tissue and perfusate) simultaneously is more advantageous. Additionally, a stability study was conducted on a subset of perfusate samples to evaluate the advantages of SPME as an on-site sample preparation tool compared to the typical sample collection approach of a traditional metabolomics workflow. The 19-h NEVLP procedure and in vivo SPME lung sampling were conducted at Toronto General Hospital (Toronto, Canada). SPME extraction of perfusate samples was performed on site at Toronto General Hospital and in the analytical laboratory at the University of Waterloo (Waterloo, Canada). LC-HRMS analysis was performed in an analytical laboratory at the University of Waterloo. The overall experimental design is illustrated in Fig. 1.

### 2.4 Animals and research ethical approval

Two Yorkshire pigs, each weighing approximately 35 kg, were used in this study. The Animal Care Committee at the Toronto General Hospital Research Institute approved the experimental protocol for domestic male Yorkshire pigs. Full ethics approval was obtained from the University Health Network Research Ethics Board and University of Waterloo Office of Research Ethics.

### 2.5 NEVLP strategy

In this study, the heart of a male Yorkshire domestic pig was excised from the heart-lung block in the left atrium (LA) after explantation. After the left atrial appendage and pulmonary artery (PA) were cannulated, the lungs were transferred to the XVIVO™ chamber (Vitrolife, Denver, CO, USA) and connected to the NEVLP circuit, which consisted of a centrifugal pump responsible for circulating perfusate (Steen™ solution; Vitrolife, Denver, CO, USA) through the circuit. The perfusate first passed through a membrane gas exchanger connected to a heat exchanger, wherein it was deoxygenated and warmed to normothermic conditions over scheduled time intervals. Once the perfusion fluid reached normothermic conditions, it was passed through a leukocyte filter and then into the lungs, which were ventilated using an ICU-type ventilator through the PA. After passing through the lungs, the perfusion fluid exited through the LA into a hard-shell reservoir adjusted to a specific height to maintain the appropriate pressure. A more detailed outline of the NEVLP procedure has been provided elsewhere [3]. The usual duration of NEVLP is 4 h; however, a prolonged schedule requiring a total of 19 h of perfusion was employed during these experiments to assess lung sustainability.

### 2.6 In vivo SPME sampling

**Fig. 2:**
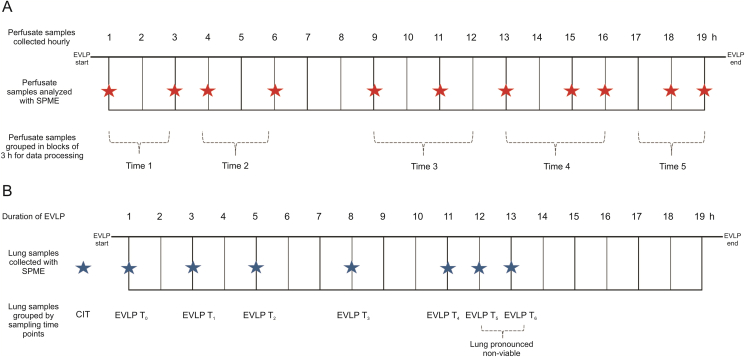
Prolonged normothermic ex vivo lung perfusion (NEVLP) conducted over 19 h. (A) Perfusate sampling schedule during NEVLP for samples prepared using thin-film microextraction (TFME). Perfusate samples were collected hourly, with red stars showing samples that were used for further analysis. Time 1 to Time 5 illustrate the blocking strategy used to group the collected samples for data processing. (B) Lung sampling schedule during NEVLP. The blue stars show the time points at which SPME fiber lung sampling events took place.

The 15 mm HLB-coated fibers were initially preconditioned in methanol:water (50:50, *V*/*V*) for at least 30 min under static conditions before exposure to the lung tissue. Once this preconditioning step was completed, the fibers were inserted into the left lung in triplicate for an extraction time of 30 min. The fibers were then removed from the lung, rinsed manually in water for 5 s, and wiped with a Kim-wipe to remove loosely adhered biological fragments. Next, the fibers were placed in empty 300 μL vials and snap frozen in dry ice for transportation to the laboratory, where they were desorbed in 137.5 μL of methanol:acetonitrile:water (40:30:30, *V*/*V*/*V*). Water was then added to the extracts to obtain a 50:50 aqueous/organic ratio, which would be more amenable to the initial chromatographic conditions. Fig. 2 illustrates the sampling schedule for the perfusate and lung samples and how the samples were grouped.

### 2.7 Data pre-treatment, data processing, and model validation

MetaboAnalyst 4.0 was the sole platform used to perform a range of multivariate and univariate statistical analyses after Pareto scaling. The original unbiased structure of the data was analyzed using principal component analysis (PCA) to evaluate the stability of the instrumental acquisition through the pooled QCs and to assess true patterns in the data structure that could be further investigated using other supervised multivariate methods. Orthogonal projections onto latent structures discriminant analysis was used to compare the two groups to identify features of differentiation, whereas partial least squares discriminant analysis (PLS-DA) was used to identify features of discrimination between three or more groups. Because the obtained data had a high degree of dimensionality, that is, the number of features was substantially larger than the number of samples, cross-validation techniques were used to confirm model reliability as well as the stability of the features deemed important by the model. To this end, quality parameters, such as goodness of fit (*R*^2^) and predictability (*Q*^2^), were assessed. Cross-validation is particularly important for supervised methods because it ensures that separation between groups is based on significant differences as opposed to noise, which is a common pitfall of these techniques. Methods such as leave-one-out cross-validation (LOOCV), *k*-fold cross-validation (CV), and permutation testing were performed within their respective limitations based on sample size. A *t*-test was used to perform univariate analysis of changes in individual features between two time groups, whereas a non-parametric analysis of variance, the Kruskal Wallis test, was used to examine these changes between three or more time groups. In addition, parametric and non-parametric univariate analyses were performed based on the limitations of the sample or feature size using a false-discovery rate-adjusted *P* value of 0.05. Because the selection of the appropriate method was based on the distribution of the data being investigated, non-parametric analysis was mostly used, as no assumptions were made regarding the data being normally distributed. Finally, heat maps were used to visualize the data and identify dysregulated features across the groups. This approach was used in the event of a failed cross-validated model or when statistically insignificant features were deemed interesting.

### 2.8 Other information

Further information outlining, including details on various sampling and data processing methods, such as the preparation of SPME fibers using a dip-coating method developed in-house [18], preparation of TFME blades [19], and the data pre-processing workflow conducted for analysis [[20], [21], [22], [23], [24], [25]], can be found in the Supplementary data S-1 to S-7.

## 3 Results and discussion

In the current study, minimally invasive in vivo SPME fibers were directly introduced into the lung to selectively extract small endogenous molecules and gain insight into the biochemical processes originating specifically in the lung tissue over the course of NEVLP (in vivo SPME). SPME fibers were also used to extract metabolites from fresh perfusate samples collected at the same time as lung sampling events (on-site ex vivo SPME). Additionally, SPME fibers were used to sample a portion of perfusate samples that were snap-frozen and subjected to further analysis (in-lab ex vivo SPME). Furthermore, another SPME format, that is, the TFME approach, was used to perform extractions on a portion of the perfusate samples that had been snap-frozen and transported to the laboratory (in-lab ex vivo TFME). This multi-faceted approach for collecting perfusate samples and the use of various devices to perform extractions yielded valuable information about metabolite alterations during NEVLP and the biochemical stability of the metabolome under typical storage and sample handling procedures. The study design allowed for the assessment of overall metabolite stability and sample storage through enabling a comparison of samples collected and sampled on site to those transported back to the laboratory. The perfusate samples collected simultaneously as lung samples during NEVLP were essential for evaluating metabolite stability and the impact of storage conditions because a portion of these perfusate samples were collected and split for on-site sampling, whereas another portion was transported for in-laboratory analysis.

### 3.1 Unsupervised multivariate analysis

**Fig. 3:**
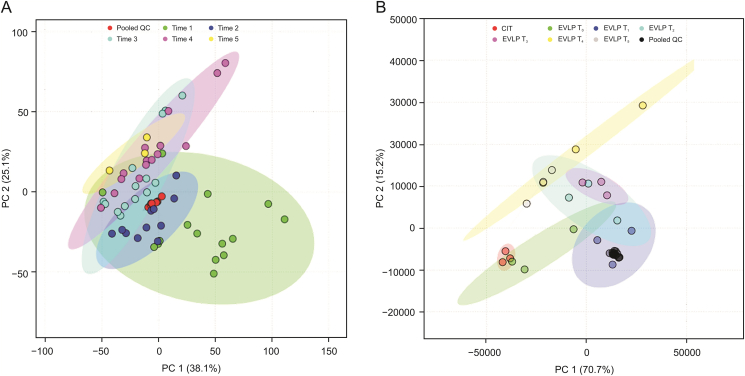
Principal component analysis (PCA). (A) PCA (PC1 38.1%, PC2: 25.1%) of the perfusate samples collected during NEVLP and prepared using TFME. Each of these time points comprises a progressive 3-h time block throughout the NEVLP process. (B) PCA (PC1: 70.7%, PC2: 15.2%) of the lungs sampled with SPME fibers. Each of these time points represents lung samples collected prior to the start of NEVLP, and 1, 3, 5, 8, 11, 12, and 13 h after the start of NEVLP. QC: quality control; CIT: cold ischemic time.

As indicated in the PCA, the pooled QCs for both sets of analyses clustered tightly in the respective plots (Fig. 3). The pooled QCs for the perfusate samples (Fig. 3A, red dots) were in the middle of the time groups, which is the most common trend observed for these types of analyses, whereas the pooled QCs for the lung samples (Fig. 3B, black dots) were closer to a particular time group. This clustering behavior might be the result of some features (metabolites) from this time group exerting a greater influence on the overall variation of the data, possibly due to a higher or lower relative abundance of certain features compared to other groups. Nevertheless, tight clustering of the pooled QCs indicates instrumental stability throughout the data acquisition, which means that the plot captures true and reliable patterns in the data structure. PCA also revealed similar patterns of distinct transitions from the initial time groups to the final time group at the end of the NEVLP for both perfusate (Fig. 3A) and lung samples (Fig. 3B). As shown in Fig. 3, the biggest differences occurred between the initial and final time groups, whereas time groups directly preceding or following one another exhibited graphical overlap. Interestingly, for both sample types, the differences between the groups became less discrete after Time 3. Furthermore, the 7-h difference in the onset of this apparent equilibrium between lung and perfusate samples may indicate that tissue sampling provides more accurate insights into when certain changes start to manifest in the organ. This 7-h time lag can be explained by differences in the processes by which metabolites diffuse from the lung to the bulk of the perfusion fluid.

The overlap between Time 4 and Time 5 clusters for perfusate samples suggests that additional hours of perfusion may neither improve nor deteriorate the state of the organ after a certain point; therefore, after 13 h of NEVLP, the rate of change appears to have reached an equilibrium. In general, the decrease in intergroup variation from the initial time group to the last also suggests that all pig lungs, regardless of their initial states, undergo the same distinct changes, as evidenced by the larger intergroup variation in Time 1 for perfusate samples.

### 3.2 Supervised multivariate analysis

**Fig. 4:**
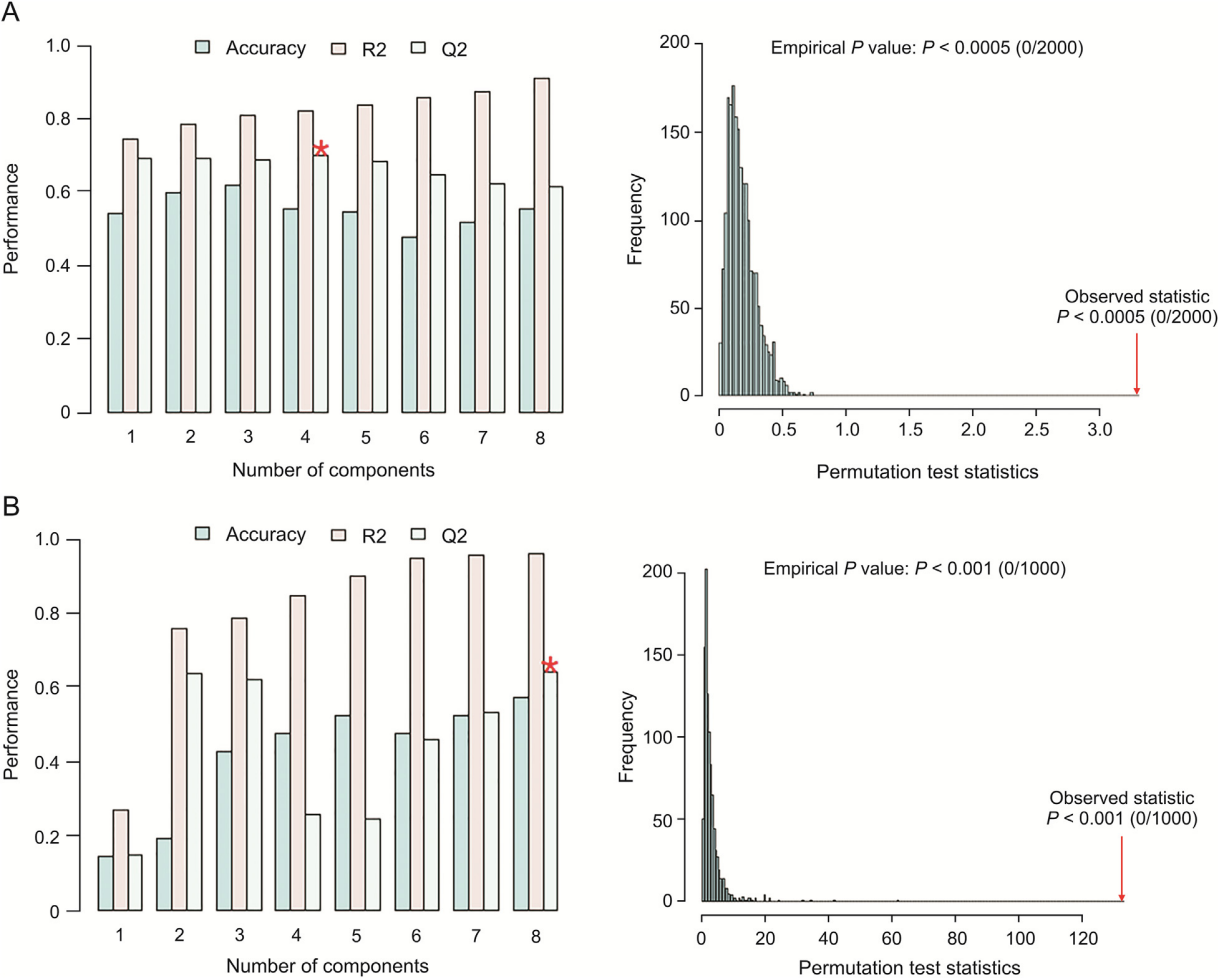
Cross validation (CV) of partial least-squares discriminant analysis models through *k*-fold CV, leave one out cross validation (LOOCV), and permutation. (A) CV for perfusate samples. On the left side, the optimized number of latent variables for a ten-fold CV is shown to be four (denoted by a red star) with quality parameters *R*^2^ = 0.82 and *Q*^2^ = 0.69. (B) CV for lung samples. On the left side, eight latent variables (denoted by a red star) were found to be optimal for an LOOCV with *R*^2^ = 0.96 and *Q*^2^ = 0.64. On the right side for both sets of data, the observed statistics for the original data set was *P* < 0.0005 and *P* < 0.001 for perfusate and lung samples, respectively (red arrows).

Both models passed permutation cross validation (2000/1000 permutations for perfusate sampled in the laboratory and lung sampled in vivo on site, respectively), yielding significant (*P* < 0.05) quality parameters for the original models (Fig. 4). Therefore, the observed statistics for the original data classifiers (groups) produced significant separation compared with the distribution of the permuted datasets. The PLS-DA model for the perfusate samples yielded quality parameters of *R*^2^ = 0.82 and *Q*^2^ = 0.69 after a 10-fold cross validation with an optimal of four latent components, whereas the lung samples yielded quality parameters of *R*^2^ = 0.96 and *Q*^2^ = 0.64 after LOOCV with an optimal of eight latent components. Although the quality parameters for the lung samples fell outside the desired range threshold (0.2 units apart), it is important to note that the number of samples per group for this data-set was quite small in comparison to the perfusate samples. Nonetheless, the *Q*^2^ for this dataset was still above the acceptable limit of 0.5. Only features with variable importance in projection (VIP) scores >1.5 for each component up until the optimized number of latent components were investigated. Therefore, 100 and 200 features were assessed for perfusate and lung samples, respectively. Table 1, Table 2 list the VIPs obtained from multivariate analysis and their respective statistical and LC-HRMS parameters for perfusate and lung samples, respectively. Specific changes in metabolites over the time period of NEVLP for lungs sampled with SPME fibers and perfusate sampled with TFME are shown in Tables S1 and S2, respectively, and discussed in Section 3.4.

**Table 1:** Variable importance in projection (VIP) > 1 obtained from validated partial least-squares discriminant analysis (PLS-DA) model for perfusate samples collected during normothermic ex vivo lung perfusion (NEVLP). The statistically significant features changing from Time 1 to Time 5, all time points inclusive, are shown (data from positive mode analysis with a mass tolerance of less than 5 ppm).

Compound classification	Feature parameters				Tentative ID
	*m*/*z*	Retention time (min)	Adduct	Average VIP score	
Notable features also found via METLIN and annotation	375.2164	13.2	[M+H]^+^	12.5	Resolvin D1 and HDOPA
	226.0807	14.3	[M+Na]^+^	3.0	Alanyl-asparagine
	324.0590	10.1	[M+H]^+^	2.4	5′-CMP and 2′,3′-cyclic UMP
	255.0870	3.75	[M+H]^+^	1.9	3-hydroxy-DL-kynurenine
	146.0039	9.3	[M+Na]^+^	1.7	Taurine
Other endogenous compounds found via METLIN	375.2164	13.2	[M+H]^+^	12.5	Prostaglandin E1
	323.1701	9.8	[M+NH_4_]^+^	8.0	Threoninyl-tryptophan
	359.2215	14.3	[M+H]^+^	5.3	4*S*-Hydroxy-8-oxo-(5*E*,9*Z*,13*Z*,16*Z*,19*Z*)-neuroprostapentaenoic acid-cyclo[7*S*,11*S*] and 17beta-hydroxyandrost-4-ene-3,11-dione propionate
	321.1849	14.5	[M+H]^+^	3.9	4,7,10,13,16-Docosapentaynoic acid
	175.1191	1.3	[M+H]^+^	2.9	d-arginine
	750.4290	13.2	[M+Na]^+^	2.5	Phosphatidylserine (12:0/20:4(5*Z*,8*Z*,11*Z*,14*Z*))

HDOPA: 11beta-hydroxy-3,20-dioxopregn-4-*en*-21-oic acid; CMP: cytidine 3′-monophosphate; UMP: uridine 3′-monophosphate.

**Table 2:** VIP > 1 obtained from validated PLS-DA model for in vivo solid phase microextraction fiber lung sampling events during NEVLP. The statistically significant features changing from EVLP *t*_0_ to EVLP *t*_5_, all time points inclusive, are shown (data from positive mode analysis).

Compound classification	Feature parameters				Tentative feature ID
	*m*/*z*	Retention time (min)	Adduct	Average VIP score	
Notable features found via METLIN and annotation	247.1077	12.3	[M+H]^+^	21.8	*N*-acetyltryptophan
	166.0863	9.7	[M+H]^+^	6.0	Phenylalanine
	226.0808	19.6	[M+Na]^+^	6.2	Alanyl-asparagine
	269.0897	12.3	[M+Na]^+^	5.7	*N*-acetyltrptophan
	205.0974	12.3	[M+H]^+^	4.4	Tryptophan
	132.1020	7.6	[M+H]^+^	3.6	l-isoleucine
	182.0813	7.6	[M+H]^+^	2.4	l-tyrosine
	147.0764	1.4	[M+H]^+^	2.0	l-glutamine
	156.0768	2.1	[M+H]^+^	1.2	Histidine
Other endogenous compounds found via METLIN	240.0965	23.0	[M+Na]^+^	13.0	*N*-alpha-acetylcitrulline, alanyl-glutamine, and 5-hydroxysebacate
	184.0577	12.3	[M+Na]^+^	3.8	dl-2-aminoadipic acid and *N*-methylglutamic acid
	137.0458	1.6	[M+H]^+^	2.9	Hypoxanthine
	270.0930	12.2	[M+NH_4_]^+^	2.1	Cysteinl-methionine
	209.0543	19.5	[M+Na]^+^	1.9	Aminomethylphosphonic acid
	269.0881	1.6	[M+H]^+^	1.9	Inosine
	156.0768	2.1	[M+NH_4_]^+^		Urocanic acid
	268.1041	7.6	[M+H]^+^	1.5	Adenosine and deoxyguanosine
			[M+Na]^+^		Glutamyl-valine

### 3.3 Univariate analysis

**Fig. 5:**
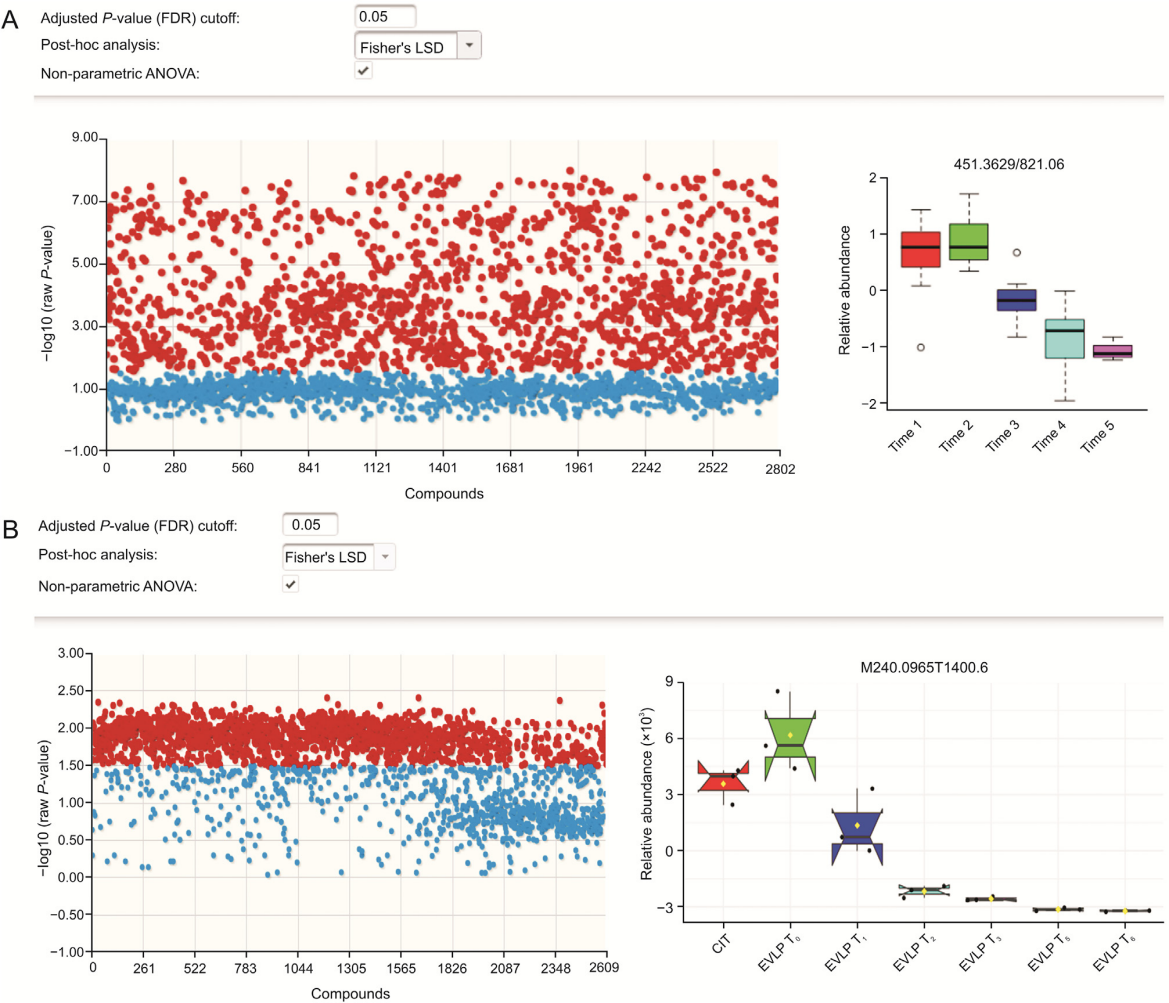
Univariate analysis using a non-parametric analysis of variance Kruskal-Wallis test for (A) perfusate samples and (B) lung samples. A false discovery rate adjusted *P* value of 0.05 was used as the cut-off for the selection of statistically significant features. Significant features are highlighted in red, whereas non-significant features are highlighted in blue. On the right side, the changing patterns in relative abundance over time of NEVLP for a single statistically significant feature is shown.

Univariate analysis was conducted using the Kruskal-Wallis test to evaluate changes in individual features throughout the NEVLP procedure. The results of this analysis yielded up to 2000 statistically significant features for perfusate samples extracted using TFME in the lab, and more than 1700 statistically significant features for lung samples extracted using the fibers during on-site in vivo SPME (Fig. 5).

### 3.4 Statistically significant metabolites and their biochemical relevance

The features that were putatively identified through annotation and the VIP > 1.5 selected from multivariate analysis (PLS-DA) indicated that it is possible to identify the presence of several endogenous metabolites related to the modulation of immune responses in a living system using in vivo SPME of lungs and on-site ex vivo SPME sampling of perfusate at specific time points during 19 h of lung perfusion. Notably, multiple bioactive lipids (oxylipins) derived from polyunsaturated fatty acids with either pro- or anti-inflammatory properties were extracted using SPME fibers over the course of NEVLP (Table S1). Neuroprostanes, which are prostaglandin-like compounds formed in vivo through the peroxidation of fatty acids (primarily docosahexaenoic acid), have been detected in lung tissue and fresh perfusate extracts during the first hours of lung perfusion. These oxygenated essential fatty acids possess potent anti-inflammatory properties and are biomarkers of oxidative stress as well as inhibitors of macrophage activity [26,27]. The presence of neuroprostanes was observed up to the fifth hour of NEVLP in lung tissue and up to the eighth hour in perfusate samples, after which the presence of other signaling molecules related to the metabolism of prostaglandins began to emerge. Specifically, the number of prostaglandin-derived compounds began to increase significantly during the third hour of NEVLP, with a large number of these compounds detected between the 11th and 12th hours (Table S1). During this period, several pro-inflammatory tissue components putatively identified as prostaglandins F3α and E2 were extracted from the lung tissue and perfusate samples. Conversely, prostaglandin D2 and its derivatives, which have been previously shown to play anti-inflammatory roles in acute lung inflammation, were also detected in the analyzed matrices during this period [28]. These results strongly indicate pro- and anti-inflammatory responses in the lungs, especially during the later hours of lung perfusion. However, since specific prostaglandins may produce various and sometimes opposite effects in different tissues, the exact role of the detected prostaglandins requires further investigation using targeted analysis of detected metabolites and their respective receptors in lung tissue.

In addition to prostaglandins, other eicosanoids, mainly leukotrienes and, to a lesser extent, lipoxins and thromboxanes, have also been observed during NEVLP. Leukotrienes are potent local mediators of immune hypersensitivity and inflammation, and the highest number of these compounds and their derivatives was detected in both lung tissue and fresh perfusate samples between the 11th and 12th hours of lung perfusion (Table S1), thus highlighting the intense immune system activity during this time point of lung perfusion. Moreover, various oxygenated lipid metabolites, including hydroxyeicosatrienoic acids (e.g., 11,12-dihydroxyeicosatrienoic acid and 5,6-dihydroxyicosa-8,11,14-trienoic acid) and epoxy fatty acids (e.g., 9,10-epoxy-12,15-octadecadienoate), were formed during lung perfusion, with significantly higher numbers being observed in lung tissue between the 11th and 12th hours of NEVLP. The findings of the present study show that in vivo SPME is more effective for sampling living systems, such as lungs undergoing NEVLP, as it can provide insights into the true oxylipin profile of the lung that cannot be achieved through ex vivo analysis of stored samples (Table S2). Overall, intense lipid peroxidation may occur under any conditions associated with pro- and anti-inflammatory responses within an organism, and elucidation of the mechanisms that drive these processes may enable the development of novel or improved targeted treatments for inflammatory diseases or processes.

Other compounds with significant anti-inflammatory and anti-apoptotic activities were also extracted using SPME fibers. Resolvins and neuroprotectins, which belong to a group of autacoids with strong in vivo immunoregulatory activity, were detected in lung tissue and perfusate samples during the first hours of lung perfusion. As previously reported, these new mediator families act locally and may strongly affect the immune system [29]. Resolvins are formed from omega-3 essential fatty acids and occur in trace amounts in tissues; however, the application of SPME probes has facilitated their successful extraction. In addition, while neuroprotectins were detected in live tissue and perfusate extracts during sampling conducted on site and in hospital, these compounds were barely detectable in the stored perfusate samples (Table S2), which may be due to their lability and/or short lifetime. These results suggest that neuroprotectin levels should be monitored in vivo. Overall, the presence of pro-inflammatory endogenous compounds may indicate ongoing inflammatory processes in the lungs during NEVLP. However, these compounds are efficiently regulated or balanced by the production of specialized mediators that stimulate anti-inflammatory responses, such as lipoxins, which have been considered as potential targets for the development of novel approaches to treating inflammation-related diseases.

In addition, the use of SPME fibers to directly sample lung tissue and perfusate samples during lung perfusion enabled the monitoring of changes in the number and composition of other endogenous molecules such as amino acids, acyl carnitines, steroid hormones, purines, and pyrimidines (Table S1). Moreover, on-site in vivo sampling of lung tissue and perfusate samples revealed the presence of oleamide, which is a fatty acyl amide (Table S1) that has previously been reported as a potential biomarker for predicting primary graft dysfunction (PGD3) during lung transplantation [1]. In contrast, this compound was scarcely detected in the stored perfusate extracts sampled through TFME, suggesting that its stability is negatively affected during storage (Table S2). Acyl carnitines, which are endogenous compounds related to fatty acid metabolism, were also extracted from lung tissue, perfusate samples collected on site during NEVLP, and stored perfusate samples. These compounds play a crucial role in β-oxidation of fatty acids, which is an alternative pathway for energy production in tissues when the supply of glucose is insufficient to maintain a normal level of energy. Elevated levels of these compounds may be related to inefficient mitochondrial importation of fatty acids through the carnitine-acylcarnitine shuttle and could indicate metabolic disorders. However, decanoylcarnitine, a metabolite of carnitine metabolism that is involved in lipid catabolism and energy production and has previously been proposed as a marker of PGD3 that could improve donor lung selection and the outcomes of lung transplantation, was present solely in stored perfusate samples collected in the first hour of NEVLP. In contrast, other acyl carnitines, including oxo- (keto-) and hydroxy-fatty acids, were detected during the entire procedure of lung perfusion, with significant increases in the number of these compounds during the third hour of NEVLP. In addition, the occurrence of malonylcarnitine in lung tissue from the third hour of NEVLP may suggest disruptions in fatty acid oxidation caused by impaired entry of long-chain acylcarnitine esters into the mitochondria.

### 3.5 Metabolite stability

**Fig. 6:**
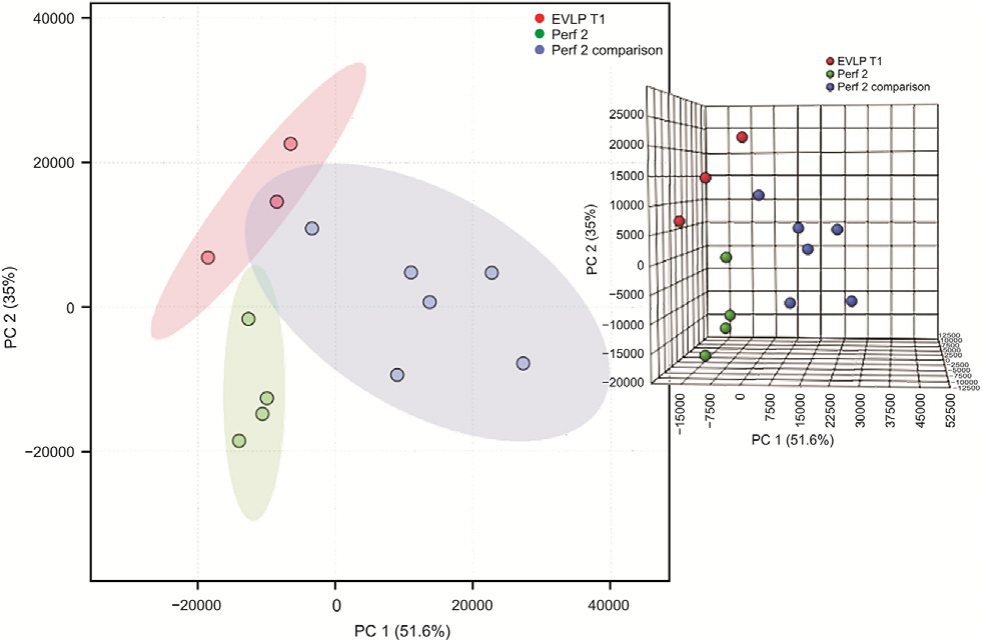
PCA (PC1 51.6%, PC2 35%, and PC3 6.5%) plot of samples collected in vivo from lungs (red), perfusate samples collected on site in the operating room (green), and perfusate samples collected, snap-frozen, and sampled ex vivo in the laboratory (blue).

**Fig. 7:**
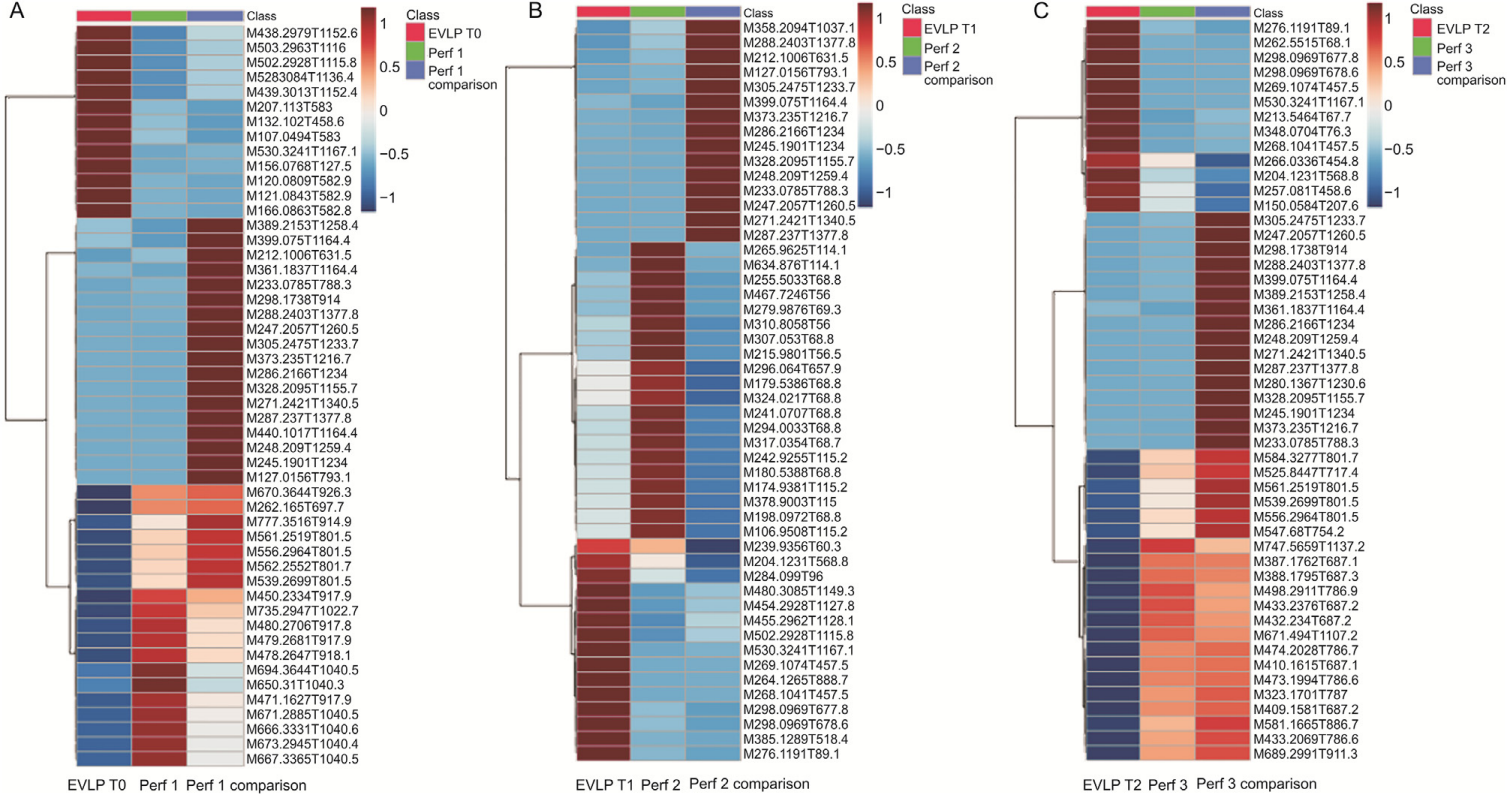
Group-averaged heat maps of samples collected in vivo from lungs (red), perfusate samples collected on site in the operating room (green), and perfusate samples collected, snap-frozen, and prepared in the laboratory (blue). Heat map for samples collected (A) after 1 h of perfusion (EVLP *t*_0_), (B) after 3 h of perfusion (EVLP *t*_1_), and (C) after 6 h of perfusion (EVLP *t*_2_). Dark red squares on the heat map indicate a high abundance of that feature in a specific group of samples, whereas dark blue indicates a low abundance.

In this multi-faceted study, the information recovered from samples collected, stored, and analyzed in the typical metabolomics workflow was compared with the information obtained through direct on-site extraction of the samples using SPME as the main sample preparation tool. Because of the dynamics of a biological system, snap freezing is often employed after sample collection to mitigate changes in the sample after it has been extracted from the system, thereby maintaining its integrity and ensuring an accurate representation of the metabolome [30]. As such, the same methods were employed to determine whether there were any significant differences between the samples that were collected and prepared with SPME fibers on site and those that were immediately snap-frozen after collection and sampled in the laboratory. Fig. 6 shows the differences between the three main sample groups, namely, lung tissue sampled on site in vivo, perfusate sampled on site at the same time as the lung tissue sampling, and perfusate that was snap-frozen and transported back to the laboratory for sampling. As previously mentioned, differences in metabolomic profiles were observed between the in vivo lung and perfusate samples (a more obvious separation can be seen in the 3D PCA plot in Fig. 6); however, there was also a distinct difference between the perfusate samples that were collected and prepared on site and those that were snap-frozen and sampled in the laboratory. Although perfusate samples were collected under the same conditions, the profiles of the relative abundance of detected features were similar for only a few features, whereas there were several upregulated or downregulated features distinguishing the on-site and in-laboratory perfusate samples (Fig. 7). This trend can be seen throughout the NEVLP process and for a wide range of compounds and features, as demonstrated during the first, third, and sixth hours of NEVLP.

Because SPME has also been reported to enable the extraction of short-lived and unstable metabolites [13,16,31], the advantages of using specific tissue sampling to complement perfusate sampling have been demonstrated in this study. Biochemical processes occurring within the lung during NEVLP have not always been detectable in perfusate samples. This may be true for short-lived or transient metabolites formed in the lungs by ongoing, rapid biochemical reactions. As presented in Fig. 7, some features and compounds highly abundant in the lung tissue were not present in either set of perfusate samples, suggesting that these features represent short-lived species or are specific only to the lung tissue. Moreover, it is possible that these features diffuse from the lung to the perfusate more slowly during perfusion and are therefore not observed in the perfusion fluid collected at the time of SPME tissue sampling. This supposition supports the observation that lung samples exhibit a more rapid transition than the metabolomic profiles observed in perfusate samples. Another aspect of interest that highlights the complementary information obtained through in vivo lung sampling and on-site sampling concerns the fundamentals of SPME, namely that it performs extraction by means of free concentration. This is demonstrated in the heat map in Fig. 7, which shows a high abundance of features in the perfusate samples collected on site, and a low abundance of these same features in the corresponding in vivo lung samples. Because SPME is a non-exhaustive technique that extracts only a portion of the free concentration being sampled, it is possible that the features with apparent low-to-zero abundance may have high binding affinity to the lung tissue, resulting in a lower free concentration available for extraction. However, because of the continuous perfusion of the organs and various diffusive processes, these features may have a higher free concentration in the perfusion fluid due to lower binding processes in comparison to a denser semi-solid biomatrix, such as the lung. These dynamics and interconnected processes emphasize the importance of performing simultaneous and complementary sampling, as well as having sample preparation tools with rapid on-site capabilities. Such tools can help provide reliable real-time pertinent biochemical information while minimizing sample handling and the number of required sample processing steps.

## 4 Conclusions

The use of prolonged (19 h) NEVLP to extend the lifetime of the lungs may induce cellular reprogramming of the organ to accommodate or adjust to the biosynthetic demands of the applied procedure. The application of in vivo SPME fibers directly into the lungs allowed the monitoring of changes occurring in the organ at the metabolite level during NEVLP. Several upregulated biochemical pathways involved in pro- and anti-inflammatory responses, as well as in lipid metabolism, were observed during extended lung perfusion, especially between the 11th and 12th hours of the procedure, and could be used as potential biomarkers of donor lung function. These changes were also observed in perfusate composition, wherein many compounds diffused from the lung during perfusion. However, unstable and/or short-lived metabolites have been extracted from the lung tissue in vivo using SPME fibers. Moreover, perfusate samples that had been stored and prepared in the laboratory according to the typical metabolomics workflow provided less complementary information about the metabolome and metabolomic profile of extracted compounds than in vivo tissue samples or perfusate samples collected in real time on site during NEVLP. As a next step, secondary and tertiary cohort metabolomic studies, as well as more targeted metabolomic approaches, should be conducted to confirm the artifact formation and other results of this untargeted metabolomic study. Furthermore, future research should monitor the metabolomic status of lungs after transplantation in recipient patients and/or employ MS/MS validation to identify potential candidate biomarkers of lung function or injury during prolonged NEVLP.

## CRediT author statement

**Nikita Looby**: Methodology, Validation, Formal analysis, Investigation, Data curation, Writing - Original draft preparation, Visualization; **Anna Roszkowska**: Methodology, Validation, Formal analysis, Investigation, Data curation, Writing - Original draft preparation, Visualization; **Aadil Ali**: Methodology, Validation, Investigation, Writing - Reviewing and Editing; **Barbara Bojko**: Conceptualization, Supervision, Writing - Reviewing and Editing, Project administration, Funding acquisition; **Marcelo Cypel**: Conceptualization, Resources, Supervision, Writing - Reviewing and Editing, Project administration, Funding acquisition; **Janusz Pawliszyn**: Conceptualization, Resources, Supervision, Writing - Original draft preparation, Project administration, Funding acquisition.

## Declaration of competing interest

The authors declare that there are no conflicts of interest.
