# Supplementary material for: Metabolomic fingerprinting of porcine lung tissue during pre-clinical prolonged ex vivo lung perfusion using in vivo SPME coupled with LC-HRMS
Source: J Pharm Anal. 2022 Jun 8;12(4):590–600. doi: 10.1016/j.jpha.2022.06.002 (PMC9463496; doi:10.1016/j.jpha.2022.06.002)
Supplement: Multimedia component 1 [file mmc1.docx]

**Supplementary Data**

**Metabolomic fingerprinting of porcine lung tissue during pre-clinical prolonged normothermic *ex vivo* lung perfusion using in vivo solid phase microextraction** **coupled to liquid chromatography-high resolution mass spectrometry**

Nikita Looby ^a, 1^, Anna Roszkowska ^a, b, 1^, Aadil Ali ^c^, Barbara Bojko ^d^, Marcelo Cypel ^c^, Janusz Pawliszyn ^a, *^

*^a^ Department of Chemistry, University of Waterloo, Waterloo, N2L 3G1, Canada.*

*^b^ Department of Pharmaceutical Chemistry, Medical University of Gdansk, 80-416, Gdansk, Poland.*

*^c^ Division of Thoracic Surgery, University Health Network, Toronto, M5G 2C4, Canada.*

*^d^ Department of Pharmacodynamics and Molecular Pharmacology, Faculty of Pharmacy, Collegium Medicum in Bydgoszcz, Nicolaus Copernicus University in Torun, 85-089, Bydgoszcz, Poland.*

^*^ Corresponding author: [janusz@uwaterloo.ca](mailto:janusz@uwaterloo.ca)

^1^ Both authors contributed equally to this work.

**S-1. SPME fiber coating methodology**

Nitinol wires (Macmaster Carr, Elmhurst, IL, USA) were measured and cut to 4 cm in length. The dip-coating procedure was carried out using a specialized software-operated dip-coating machine that had been developed in-laboratory, and was consistent with the protocol reported by Gómez-Ríos et al. [18]. Briefly, the protocol used in this study consisted of the following steps: first, a slurry of 10% (*m*/*V*) of 5 µm of hydrophilic-lipophilic balanced (HLB) particles suspended in 7% PAN was prepared; next, the fibers were dipped in the slurry to the desired length and then cured for 1 min at 125 °C; finally, the dipping/curing process was repeated until the final fiber coating dimensions measured 15 mm in length and 40 µm in thickness.

**S-2. TFME blade coating methodology**

Thin-film microextraction devices were prepared according to a well established spray coating method developed in-laboratory and reported by Mirnaghi et al. [19]. First, the stainless-steel blades were etched for 1 h in concentrated hydrochloric acid and then thoroughly rinsed in deionized water. After rinsing, the blades were dried in an oven for 45 min. Next, a slurry consisting of 10% (*m*/*V*) 45−60 µm HLB particles with 25% (*V*/*V*) DMF in PAN solution (7%) was prepared and mixed thoroughly in a flask-type sprayer. The slurry was then sprayed onto each stainless-steel comb individually using a maximum flow of nitrogen gas. After both sides of the comb had been coated, it was cured for 1 min at 150 °C. The final thin-film microextraction (TFME) coating measured 1 cm in length and 0.5 mm in thickness.

**S-3. The multifaceted approach**

Perfusate was collected periodically during normothermic ex vivo lung perfusion (NEVLP) and split into three portions: portion one was sampled on-site during NEVLP using solid phase microextraction (SPME) HLB fibers that were similar to those used to sample the lung tissue; portion two was snap frozen in dry ice, transported and stored at –80 °C until later sampling in the lab with SPME HLB fibers; and portion three was snap frozen and stored under the same conditions as portion two until later sampling in the lab with the HLB TFME device. The perfusate samples were processed in this way in order to: 1) evaluate the differences in the information obtained from the tissue and parallel perfusion fluid samples; 2) investigate the changes in the perfusate samples and compare them to the metabolic results reported in prior biomarker studies; 3) assess whether metabolic changes had occurred in the perfusate samples due to the sample storage procedure, which was consistent with that used in a typical metabolomics workflow (i.e., collecting and storing samples for analysis at a later date); and 4) evaluate the analytical differences between the two types of SPME devices

**S-4. Lung and perfusate sampling with SPME fiber**

Lung sampling was performed during cold ischemia (CIT) before the start of NEVLP, and at 1, 3, 5, 8, 11, 12, and 13 h after the start of NEVLP (denoted by blue stars in Fig. 2B). In addition, perfusate samples were collected hourly by clinicians and separated into three portions for analysis. The first portion (1500 µL) was sampled on site (in-hospital) in triplicate (3−300 µL portions) for 30 min under static conditions using the SPME fibers and the above-described protocol for rinsing, storage, and desorption. On-site SPME fiber sampling was only performed using perfusate samples that were acquired at the same time as the lung sampling. The second portion (1500 µL) was snap frozen in dry ice for transportation to the laboratory. These samples were later prepared using SPME fibers, at the same volume (300 µL) as used in-hospital in triplicate and using the same sampling protocol as described earlier in this section. Only perfusate samples obtained during lung sampling were used for the in-laboratory extractions with the SPME fibers. TFME was used to perform extractions on the third portion of perfusate samples. This process is described in more detail below.

**S-5. Perfusate sampling with TFME**

Perfusate samples were collected by clinicians hourly during the 19 h EVLP procedure and immediately snap frozen in liquid nitrogen as per institutional practice at Toronto General Hospital. These samples, which were later transported to the laboratory in dry ice, constituted the third portion of perfusate samples. From the 19 perfusate samples collected per animal (7 animals in total), a few samples at specific time points (denoted by red stars in Fig. 2) were selected from each animal for further analysis, with 550 µL of each selected sample being placed into a 96-well plate. Prior to performing the extractions, the TFME brush device, which is compatible with 96-well plates, was preconditioned for 30 min in 50:50 (*V*/*V*) methanol/water at 1,500 r/min followed by a quick 10 s rinse in water at 1500 r/min. After preconditioning, the TFME brush device was inserted into the samples for 30 min at 1500 r/min and a temperature of 25 °C. The blades were then rinsed in water for 10 s at 1500 rpm and desorbed in 550 µL of methanol:acetonitrile:water (40:30:30, *V*/*V*/*V*) for 1 h at 1500 r/min. A Concept-96 autosampler (PAS technologies) was used to automate the above-described SPME sample preparation process. This method was used to perform extractions from perfusate samples obtained from a total of 7 pigs subjected to NEVLP.

**S-6. Data pre-processing**

The raw LC-MS data files were first converted into mzXML files via MSConvert [20] before being pre-processed using the XCMS software package [21] in RStudio with an in-laboratory developed script [22]. The IPO package [23] used the pooled quality control (QC) samples to optimize the data pre-processing parameters used to perform peak alignment, retention time correction, normalization, peak filling, and peak picking. The paired mass difference function was also employed to reduce the number of redundant features that might be investigated [24]. This function was used in combination with other filtering methods to remove features with: 1) low spray stability or high variation in the pooled QCs (features in the pooled QCs with RSDs > 30% were eliminated); 2) signal intensities that were similar to those of the blank fibers and blank blades (features with a pooled QC:blank fiber/blank blade ratio of < 5 were eliminated); 3) a similar signal intensity to the blank solvent (features with a pooled QC:blank solvent ratio of < 5 were eliminated); and d) a similar signal intensity to the pure steen solution (features with a pooled QC:steen solution ratio at a baseline of < 5 were eliminated). These filtering methods substantially reduced the total number of features included in subsequent chemometric analyses, thereby improving data manageability and mitigating potential artefacts. The extracted peaks were annotated using the xMSannotator Integrative Scoring Algorithm in conjunction with the human metabolome database [25]. For metabolite profiling, unique and multiple features (metabolites) with medium-to-high confidence annotations were selected. xMSannotator annotated these features based on their intensity profiles, retention times, mass defect, and the isotope/adduct patterns of their peaks. For high-confidence matches, the following requirements are satisfied: a non-zero score for database matching, user specified required adducts are present (i.e. [M+H] or [M−H] for positive and negative modes respectively), N, O, P, S/C ratio checks, hydrogen/carbon ratio check, abundance ratio checks for isotopes, multimers, and multiply charged adducts. For medium-confidence matches, pathway level correlation is satisfied.

**S-7. Considerations prior to data analysis**

A total of 2 pigs underwent lung sampling during NEVLP. the first pig, the lung was sampled in triplicate at all time points, while for the second case, lung was only sampled during the first two sampling points (CIT and NEVLP at 1 h) and at the end of NEVLP (NEVLP at 12 h) when the lung was pronounced non-viable. The relative lack of lung sampling for the second case, as well as the lack of cases for this aspect of the study (lung sampling with SPME fibers), was due to unexplained and extensive lung damage sustained during NEVLP.

The lung samples from both pig cases were grouped based on the time point at which they were collected during NEVLP. For example, samples collected during cold ischemic time for both pig 1 and pig 2 (CIT) were assigned to one time group, while samples collected in the first hour of NEVLP, NEVLP at 1h (EVLP *t*_0_ as outlined in Fig. 2B), were assigned to a separate time group, and so forth. See Fig. 2B for further details on the grouping of lung samples collected via SPME fibers. Many of the SPME fiber time groups only contained a small number of samples (3−6 samples, 3 technical replicates, 2 biological replicates) since the number of lungs sampled with these devices was small (2 cases), and the number of lungs sampled to the completion of the NEVLP procedure was even smaller (1 case).

Although perfusate samples were collected on an hourly basis throughout the 19 h NEVLP process for 7 pigs, they were ultimately processed in blocks/groups of 3 h due to the preliminary nature of the study. For example, group 1, otherwise referred to as Time 1 in Fig. 2A, contained perfusate samples from 7 cases that were collected within 1−3 h after the start of NEVLP. While this data processing design limits the capturing of possibly important short-lived species, this compromise was implemented for improved data manageability.

**Tables**

**Table S1.** Comparison of detected features in lung and perfusate samples extracted with the use of solid phase microextraction (SPME) fibers. Unique and multiple features with medium-to-high confidence matches annotated by human metabolome database (HMDB) were selected. The annotation was based on intensity profiles, retention time, mass defect, and isotope/adduct patterns of peaks. In high confidence match, non-zero xMSannotator multistage score, required adducts, N, O, P, S/C ratio check, hydrogen/carbon ratio check, abundance ratio checks for isotopes, multimers and multiply charged adducts are satisfied; in medium confidence match, pathway level correlation is satisfied. LUNG_in vivo: in vivo extraction from lungs with the use of SPME fibers; PERFUSATE_on site: on site extraction from fresh perfusate samples with the use of SPME fibers collected at particular time-points compatible with lung sampling time-points.

|  | **CIT** | **1st h** | | | | **3rd h** | | **5th h** | | | | | **8th h** | | | | **11th-12th h** | | | **13th-14th h** | | |  |
| --- | --- | --- | --- | --- | --- | --- | --- | --- | --- | --- | --- | --- | --- | --- | --- | --- | --- | --- | --- | --- | --- | --- | --- |
|  | LUNG in vivo | LUNG in vivo | | PERFUSATE on site | | LUNG  in vivo | PERFUSATE on site | LUNG  in vivo | | PERFUSATE on site | | | LUNG  in vivo | | PERFUSATE on site | | LUNG  in vivo | | PERFUSATE on site | LUNG  in vivo | PERFUSATE on site | |  |
| **AMINO ACIDS** | N-Acetylhistamine | N-Acetylhistamine | | L-Phenylalanine | | L-Histidine | N-Acetylhistamine | L-Histidine | | N-Acetylhistamine | | | L-Histidine | | L-Histidine | | N-Acetylhistamine | | L-Tryptophan | N-Acetylhistamine | L-Histidine | |  |
|  | DL-Glutamate | L-Cystine | | L-Tryptophan | | N-Acetylhistamine | N-acetyltryptophan | L-Tryptophan | | L-Tryptophan | | | L-Tryptophan | | N-Acetylhistamine | | L-Tryptophan | | L-Kynurenine | Kynurenic acid | N-Acetylhistamine | |  |
|  | L-Glutamic acid | L-Asparagine | | Kynurenic acid | | L-Tryptophan | Indoleacetyl glutamine | L-Kynurenine | | Indoleacetyl glutamine | | | Indoleacetyl glutamine | | L-Glutamine | | Indoleacetyl glutamine | | L-Glutamine | L-Glutamine | L-Kynurenine | |  |
|  | L-4-Hydroxyglutamate semialdehyde | L-Threonine | | L-Leucine | | L-Kynurenine | L-Glutamic acid | L-Glutamic acid | | L-Glutamine | | | Quinaldic acid | | L-Glutamic acid | | Quinaldic acid | | L-Glutamic acid | L-Glutamic acid | Indoleacetyl glutamine | |  |
|  | N-Methyl-D-aspartic acid | L-Proline | | L-Isoleucine | | Indoleacetyl glutamine | L-Glutamine | L-Glutamic acid | | L-Glutamic acid | | | L-Glutamine | | L-Tryptophan | | L-Kynurenine | | Pyroglutamic acid | L-Phenylalanine | 2-Aminomuconic acid | |  |
|  | O-Acetylserine | L-Glutamine | | L-Methionine | | L-Glutamic acid | Pyroglutamic acid | Pyroglutamic acid | | Pyroglutamic acid | | | L-Lysine | | N-acetyltryptophan | | L-Glutamic acid | | L-Threonine | L-Methionine | L-Glutamine | |  |
|  | N-Undecanoylglycine | L-Arginine | | L-Arginine | | Pyroglutamic acid | L-Valine | D-Arginine | | L-Proline | | | L-Phenylalanine | | L-Kynurenine | | D-Glutamic acid | | L-Proline | L-Cystine | Pyroglutamic acid | |  |
|  | Tetracosanoylglycine | L-Glutamic acid | | L-Cystine | | Gamma-Glutamyltyrosine | L-Cystine | L-Valine | | L-Phenylalanine | | | Ne,Ne dimethyllysine | | Indoleacetyl glutamine | | L-4-Hydroxyglutamate semialdehyde | | L-Cystine | L-Lysine | L-Lysine | |  |
|  | N-Nonanoylglycine | 5-Aminopentanamide | | L-Proline | | L-Leucine | L-Lysine | L-Leucine | | Capryloylglycine | | | L-Leucine | | L-Leucine | | O-Acetylserine | | N(6)-(Octanoyl)lysine | L-Proline | L-Phenylalanine | |  |
|  |  | Creatine | | L-Tyrosine | | L-Isoleucine | Creatinine | L-Arginine | | Tridecanoylglycine | | | L-Isoleucine | | L-Phenylalanine | | N-Acetylserine | | L-Methionine | L-Tyrosine | L-Leucine | |  |
|  |  | Creatinine | | L-Glutamic acid | | L-Phenylalanine | N-Acryloylglycine | L-Isoleucine | | N-Undecanoylglycine | | | L-Methionine | | Pyroglutamic acid | | Creatinine | | L-Arginine | Creatine | L-Isoleucine | |  |
|  |  | N-Decanoylglycine | | L-alpha-glutamyl-L-hydroxyproline | | D-Serine | Tridecanoylglycine | L-Phenylalanine | | N-Nonanoylglycine | | | L-Cystine | | L-Isoleucine | | N-Undecanoylglycine | | Creatine | N-Undecanoylglycine | N-(5-Methyl-3-oxohexyl)alanine | |  |
|  |  | N-Nonanoylglycine | | Creatine | | L-Methionine | N-Decanoylglycine | NNAL-N-glucuronide | | Palmitoylglycine | | | L-Asparagine | | L-Methionine | | Tridecanoylglycine | | Creatinine | N-Acryloylglycine | L-Arginine | |  |
|  |  | Palmitoylglycine | | N-Undecanoylglycine | | L-Cystine | Palmitoylglycine | N-Acetyl-S-(N-methylcarbamoyl)cysteine | | N-Acryloylglycine | | | L-Threonine | | L-Arginine | | Tricosanoylglycine | | N-Undecanoylglycine | Tridecanoylglycine | L-Tyrosine | |  |
|  |  |  | | Tridecanoylglycine | | L-Threonine | N-Undecanoylglycine | N-Undecanoylglycine | | Tridecanoylglycine | | | Creatine | | L-Cystine | | Tetracosanoylglycine | | Valproylglycine | Pristanoylglycine | Tetracosanoylglycine | |  |
|  |  |  |  | Tetracosanoylglycine | | L-Tyrosine | Tridecanoylglycine | Valproylglycine | | N-Decanoylglycine | | | Creatinine | | L-Lysine | | N-Nonanoylglycine | | 5-L-Glutamylglycine | N-Nonanoylglycine | N-Acryloylglycine | |  |
|  |  |  |  | Pristanoylglycine | | L-Valine |  | Tridecanoylglycine | | Valproylglycine | | | Tetracosanoylglycine | | L-Threonine | | Palmitoylglycine | | Capryloylglycine | N-Lauroylglycine | Capryloylglycine | |  |
|  |  |  |  | N-Nonanoylglycine | | 5-Oxoprolinate |  | Tetracosanoylglycine | |  | | | N-Undecanoylglycine | | L-Proline | |  | | Tridecanoylglycine | N-Decanoylglycine | N-Undecanoylglycine | |  |
|  |  |  |  | N-Lauroylglycine | | Creatinine |  | Stearoylglycine | |  |  |  | N-Decanoylglycine | | Creatine | |  |  | Tetracosanoylglycine | Tridecanoylglycine | N-Nonanoylglycine | |  |
|  |  |  |  | Dimethylglycine | | Creatine |  | N-Nonanoylglycine | |  |  |  | Tridecanoylglycine | | N-Undecanoylglycine | |  |  | Pristanoylglycine |  | N-Nonanoylglycine | |  |
|  |  |  |  |  | | N-Acryloylglycine |  | N-Lauroylglycine | |  |  |  | Pristanoylglycine | | N-Acryloylglycine | |  |  | N-Nonanoylglycine |  | Palmitoylglycine | |  |
|  |  |  |  |  |  | Tetracosanoylglycine |  | N-Decanoylglycine | |  |  |  | N-Nonanoylglycine | | Tridecanoylglycine | |  |  | N-Lauroylglycine |  |  | |  |
|  |  |  |  |  |  | Pristanoylglycine |  | Palmitoylglycine | |  |  |  | N-Lauroylglycine | | Tetracosanoylglycine | |  |  | N-Decanoylglycine |  |  |  |  |
|  |  |  |  |  |  | N-Undecanoylglycine |  | N-Acryloylglycine | |  |  |  | Palmitoylglycine | | Stearoylglycine | |  |  | Palmitoylglycine |  |  |  |  |
|  |  |  |  |  |  | N-Nonanoylglycine |  |  | |  |  |  | Capryloylglycine | | N-Nonanoylglycine | |  |  | N-Acryloylglycine |  |  |  |  |
|  |  |  |  |  |  | Palmitoylglycine |  |  |  |  |  |  | Tridecanoylglycine | | N-Lauroylglycine | |  |  |  |  |  |  |  |
|  |  |  |  |  |  | Tridecanoylglycine |  |  |  |  |  |  | N-Decanoylglycine | | N-Decanoylglycine | |  |  |  |  |  |  |  |
|  |  |  |  |  |  | N-Decanoylglycine |  |  |  |  |  |  | 5-L-Glutamylglycine | | Dimethylglycine | |  |  |  |  |  |  |  |
|  |  |  |  |  |  |  |  |  |  |  |  |  |  | | Tridecanoylglycine | |  |  |  |  |  |  |  |
|  |  |  |  |  |  |  |  |  |  |  |  |  |  |  | 5-L-Glutamylglycine | |  |  |  |  |  |  |  |
| **PEPTIDES** |  |  | | Phenylalanyl-Arginine | |  | Arginyl-Phenylalanine |  | | Phenylalanyl-Arginine | | | Phenylalanyl-Arginine | | Phenylalanyl-Arginine | | Arginyl-Arginine | | Aspartyl-Alanine | Phenylalanyl-Arginine |  | |  |
|  |  |  |  | Arginyl-Phenylalanine | |  |  |  |  | Arginyl-Phenylalanine | | | Aspartyl-Alanine | | Aspartyl-Alanine | |  | | Alanyl-Aspartate | Arginyl-Phenylalanine |  |  |  |
|  |  |  |  |  | |  |  |  |  | L-Cysteinylglycine disulfide | | | Arginyl-Phenylalanine | | Arginyl-Phenylalanine | |  |  | L-Cysteinylglycine disulfide |  |  |  |  |
|  |  |  |  |  |  |  |  |  |  |  | | | Alanyl-Aspartate | | Alanyl-Aspartate | |  |  |  |  |  |  |  |
|  |  |  |  |  |  |  |  |  |  |  |  |  | Valproylglycine | |  | |  |  |  |  |  |  |  |
|  |  |  |  |  |  |  |  |  |  |  |  |  | Gamma-Glutamyltyrosine | |  |  |  |  |  |  |  |  |  |
|  |  |  |  |  |  |  |  |  |  |  |  |  | L-Cysteinylglycine disulfide | |  |  |  |  |  |  |  |  |  |
| **ACYLCARNITINES** | 6-Keto-decanoylcarnitine | 6-Keto-decanoylcarnitine | | Propionylcarnitine | | 6-Keto-decanoylcarnitine | Propionylcarnitine | 6-Keto-decanoylcarnitine | | L-Acetylcarnitine | | | 6-Keto-decanoylcarnitine | | 6-Keto-decanoylcarnitine | | 12-Hydroxy-12-octadecanoylcarnitine | | 12-Hydroxy-12-octadecanoylcarnitine | 6-Keto-decanoylcarnitine | 6-Keto-decanoylcarnitine | |  |
|  | 12-Hydroxy-12-octadecanoylcarnitine | L-Acetylcarnitine | | Arachidonyl carnitine | | L-Acetylcarnitine | Arachidonyl carnitine |  | | Propionylcarnitine | | | Malonylcarnitine | | Propionylcarnitine | | L-Acetylcarnitine | | L-Acetylcarnitine | L-Acetylcarnitine | L-Acetylcarnitine | |  |
|  |  |  | |  | | Malonylcarnitine | Malonylcarnitine |  |  |  | | |  | | L-Acetylcarnitine | | L-Carnitine | | Malonylcarnitine |  | Malonylcarnitine | |  |
|  |  |  |  |  |  | L-Palmitoylcarnitine | L-Acetylcarnitine |  |  |  |  |  |  |  |  | |  | | Arachidonyl carnitine |  |  | |  |
|  |  |  |  |  |  | L-Carnitine |  |  |  |  |  |  |  |  |  |  |  |  |  |  |  |  |  |
| **AUTACOIDS** | Resolvin D1 |  | | Resolvin D5 | | Resolvin D1 | Resolvin D1 | Resolvin D1 | | Resolvin D1 | | |  | | Resolvin D1 | | Resolvin D5 | | Resolvin D5 | Resolvin D5 |  | |  |
|  | Resolvin D2 |  |  | Neuroprotectin D1 | | Resolvin D2 | Resolvin D2 | Resolvin D2 | | Resolvin D2 | | |  |  | Resolvin D2 | | Neuroprotectin D1 | | Neuroprotectin D1 | Neuroprotectin D1 |  |  |  |
|  |  |  |  |  | |  | Resolvin D5 | Resolvin D5 | |  | | |  |  | Resolvin D5 | |  | |  |  |  |  |  |
|  |  |  |  |  |  |  | Neuroprotectin D1 | Neuroprotectin D1 | |  |  |  |  |  | Neuroprotectin D1 | |  |  |  |  |  |  |  |
| **PROSTAGLANDINS** | 7-hydroxy-D4-neuroprostane |  |  | | 7-hydroxy-D4-neuroprostane | | 7-hydroxy-D4-neuroprostane | | 7-hydroxy-D4-neuroprostane | | 7-hydroxy-D4-neuroprostane |  | | 4-hydroxy-D4-neuroprostane | | Prostaglandin B1 | | Levuglandin E2 | |  | |  | |
|  | 4-hydroxy-D4-neuroprostane |  |  |  | 4-hydroxy-D4-neuroprostane | | 4-hydroxy-D4-neuroprostane | | 4-hydroxy-D4-neuroprostane | | 4-hydroxy-D4-neuroprostane |  |  | 20-hydroxy-E4-neuroprostane | | Prostaglandin A1 | | 13,14-Dihydro-15-keto-PGD2 | |  |  |  |  |
|  | 20-hydroxy-E4-neuroprostane |  |  |  | 20-hydroxy-E4-neuroprostane | | 20-hydroxy-E4-neuroprostane | | 20-hydroxy-E4-neuroprostane | | 20-hydroxy-E4-neuroprostane |  |  | 17-hydroxy-E4-neuroprostane | | 23-Dinor-6-keto-prostaglandin F1 a | | 11b-PGE2 | |  |  |  |  |
|  | 17-hydroxy-E4-neuroprostane |  |  |  | 17-hydroxy-E4-neuroprostane | | 17-hydroxy-E4-neuroprostane | | 17-hydroxy-E4-neuroprostane | | 17-hydroxy-E4-neuroprostane |  |  | 14-hydroxy-E4-neuroprostane | | 8-iso-PGA1 | | 8-iso-PGF3a | |  |  |  |  |
|  | 14-hydroxy-E4-neuroprostane |  |  |  | 14-hydroxy-E4-neuroprostane | | 14-hydroxy-E4-neuroprostane | | 14-hydroxy-E4-neuroprostane | | 14-hydroxy-E4-neuroprostane |  |  | 7-hydroxy-D4-neuroprostane | | Prostaglandin C1 | | Prostaglandin F3a | |  |  |  |  |
|  |  |  |  |  | 5,6-Dihydroxyprostaglandin F1a | |  | | PGF2a ethanolamide | | 13,14-Dihydro PGF-1a |  |  |  | | 9-Deoxy-delta12-PGD2 | | Prostaglandin D2 | |  |  |  |  |
|  |  |  |  |  |  | |  |  |  | | 13,14-Dihydro-15-keto-PGD2 |  |  |  |  |  | | Prostaglandin H2 | |  |  |  |  |
|  |  |  |  |  |  |  |  |  |  |  | 11b-PGE2 |  |  |  |  |  |  | Prostaglandin I2 | |  |  |  |  |
|  |  |  |  |  |  |  |  |  |  |  | 8-isoprostaglandin E2 |  |  |  |  |  |  | Prostaglandin E2 | |  |  |  |  |
|  |  |  |  |  |  |  |  |  |  |  | 8-iso-15-keto-PGF2a |  |  |  |  |  |  | 8-isoprostaglandin E2 | |  |  |  |  |
|  |  |  |  |  |  |  |  |  |  |  | 15-Keto-prostaglandin F2a |  |  |  |  |  |  | 8-iso-15-keto-PGF2a | |  |  |  |  |
|  |  |  |  |  |  |  |  |  |  |  | 13,14-Dihydro-15-keto-PGE2 |  |  |  |  |  |  | 15-Keto-prostaglandin F2a | |  |  |  |  |
|  |  |  |  |  |  |  |  |  |  |  | 8-iso-PGF3a |  |  |  |  |  |  | 13,14-Dihydro PGF-1a | |  |  |  |  |
|  |  |  |  |  |  |  |  |  |  |  | Prostaglandin F3a |  |  |  |  |  |  | (5Z)-(15S)-11alpha-Hydroxy-9,15-dioxoprostanoate | |  |  |  |  |
|  |  |  |  |  |  |  |  |  |  |  | Prostaglandin D2 |  |  |  |  |  |  | (13E)-11a-Hydroxy-9,15-dioxoprost-13-enoic acid | |  |  |  |  |
|  |  |  |  |  |  |  |  |  |  |  | Prostaglandin H2 |  |  |  |  |  |  |  | |  |  |  |  |
|  |  |  |  |  |  |  |  |  |  |  | PGF2a ethanolamide |  |  |  |  |  |  |  |  |  |  |  |  |
|  |  |  |  |  |  |  |  |  |  |  | Prostaglandin E2 |  |  |  |  |  |  |  |  |  |  |  |  |
| **THROMBOXANES** |  |  | |  | |  |  |  | | Thromboxane A2 | | |  | |  | | 2,3-Dinor-TXB2 | | Thromboxane A2 |  |  | |  |
| **LEUKOTRIENES** | Leukotriene C5 |  | | Leukotriene E4 | | Omega-Carboxy-trinor-leukotriene B4 | Leukotriene E4 | Omega-Carboxy-trinor-leukotriene B4 | | Omega-Carboxy-trinor-leukotriene B4 | | | 12-Oxo-20-trihydroxy-leukotriene B4 | | Leukotriene E4 | | 67-dihydro-12-epi-LTB4 | | 67-dihydro-12-epi-LTB4 | 6,7-dihydro-12-epi-LTB4 | Leukotriene E4 | |  |
|  | Leukotriene E4 |  |  |  | | 10,11-dihydro-20-trihydroxy-leukotriene B4 | 67-dihydro-12-epi-LTB4 | Leukotriene C5 | | 10,11-dihydro-20-trihydroxy-leukotriene B4 | | | 1011-dihydro-20-trihydroxy-leukotriene B4 | |  | | 12-Keto-tetrahydro-leukotriene B4 | | 10,11-dihydro-leukotriene B4 | 10,11-dihydro-leukotriene B4 |  | |  |
|  |  |  |  |  |  |  | 12-Oxo-20-trihydroxy-leukotriene B4 |  | | 20-Hydroxy-leukotriene B4 | | | Leukotriene E4 | |  |  | Omega-Carboxy-trinor-leukotriene B4 | | 20-Hydroxy-leukotriene B4 | 12-Keto-tetrahydro-leukotriene B4 |  |  |  |
|  |  |  |  |  |  |  | 1011-dihydro-leukotriene B4 |  |  |  | | | Leukotriene C5 | |  |  | Leukotriene C5 | | Omega-Carboxy-trinor-leukotriene B4 | Leukotriene E4 |  |  |  |
|  |  |  |  |  |  |  | 12-Keto-tetrahydro-leukotriene B4 |  |  |  |  |  |  | |  |  | Leukotriene E4 | | Leukotriene C5 |  |  |  |  |
|  |  |  |  |  |  |  |  |  |  |  |  |  |  |  |  |  | 67-dihydro-5-oxo-12-epi-LTB4 | | 10,11-dihydro-20-trihydroxy-leukotriene B4 |  |  |  |  |
|  |  |  |  |  |  |  |  |  |  |  |  |  |  |  |  |  | 1011-dihydro-12-oxo-LTB4 | | 12-Keto-tetrahydro-leukotriene B4 |  |  |  |  |
|  |  |  |  |  |  |  |  |  |  |  |  |  |  |  |  |  | 12(S)-Leukotriene B4 | | Leukotriene E4 |  |  |  |  |
|  |  |  |  |  |  |  |  |  |  |  |  |  |  |  |  |  | 6-trans-12-epi-Leukotriene B4 | |  |  |  |  |  |
|  |  |  |  |  |  |  |  |  |  |  |  |  |  |  |  |  | 6-trans-Leukotriene B4 | |  |  |  |  |  |
|  |  |  |  |  |  |  |  |  |  |  |  |  |  |  |  |  | Leukotriene B4 | |  |  |  |  |  |
| **LIPOXINS** |  |  | |  | |  |  |  | | 15-Epi-lipoxin A4 | | |  | |  | |  | | 15-Epi-lipoxin A4 |  |  | |  |
|  |  |  |  |  |  |  |  |  |  | 13,14-Dihydro-15-oxo-lipoxin A4 | | |  |  |  |  |  |  | 13,14-Dihydro-15-oxo-lipoxin A4 |  |  |  |  |
|  |  |  |  |  |  |  |  |  |  | Lipoxin B4 | | |  |  |  |  |  |  | Lipoxin A4 |  |  |  |  |
|  |  |  |  |  |  |  |  |  |  | Lipoxin A4 | | |  |  |  |  |  |  | Lipoxin B4 |  |  |  |  |
| **OTHER OXYLIPINS** |  |  | | 9,10-DiHODE | | 9,10,13-TriHOME | 9,10-DiHODE | 9,10,13-TriHOME | | 9,10-DiHODE | | | 9,10-DiHODE | | 15,16-DiHODE | | 9,10,13-TriHOME | | 9,10-DiHODE | 9,10-DiHODE |  | |  |
|  |  |  |  | 15,16-DiHODE | | 9,12,13-TriHOME | 15,16-DiHODE | 9,12,13-TriHOME | | 15,16-DiHODE | | | 15,16-DiHODE | | 12,13-DiHODE | | 9,12,13-TriHOME | | 15,16-DiHODE | 15,16-DiHODE |  |  |  |
|  |  |  |  | 12,13-DiHODE | | 12(13)Ep-9-KODE | 12,13-DiHODE |  | | 12,13-DiHODE | | | 12,13-DiHODE | | 9,10,13-TriHOME | | 5,6-DHET | | 12,13-DiHODE | 12,13-DiHODE |  |  |  |
|  |  |  |  | 12(13)Ep-9-KODE | |  | 9,10,13-TriHOME |  |  | 9,10,13-TriHOME | | | 9(S)-HPODE | | 9,12,13-TriHOME | | 8,15-DiHETE | | 9,10,13-TriHOME | 9,12,13-TriHOME |  |  |  |
|  |  |  |  | 9(S)-HPODE | |  | 9,12,13-TriHOME |  |  | 9,12,13-TriHOME | | | 12(13)Ep-9-KODE | | 9-HOTE | | 5,15-DiHETE | | 9,12,13-TriHOME | 9(S)-HPODE |  |  |  |
|  |  |  |  |  | |  | 9(S)-HPODE |  |  | 9(S)-HPODE | | |  | | 13-HOTE | | 17,18-DiHETE | | 9(S)-HPODE | 12(13)Ep-9-KODE |  |  |  |
|  |  |  |  |  |  |  | 5,6-DHET |  |  | 12(13)Ep-9-KODE | | |  |  | 9(S)-HPODE | | 14,15-DiHETE | | 5,6-DHET | 5,6-DHET |  |  |  |
|  |  |  |  |  |  |  | 11,12-DiHETrE |  |  |  | | |  |  | 9(10)-EpODE | | 15H-11,12-EETA | | 11,12-DiHETrE | 11,12-DiHETrE |  |  |  |
|  |  |  |  |  |  |  | 8,9-DiHETrE |  |  |  |  |  |  |  | A-12(13)-EpODE | | 11H-14,15-EETA | | 8,9-DiHETrE | 8,9-DiHETrE |  |  |  |
|  |  |  |  |  |  |  | 14,15-DiHETrE |  |  |  |  |  |  |  | 9-OxoODE | | 11,12-DiHETrE | | 14,15-DiHETrE | 14,15-DiHETrE |  |  |  |
|  |  |  |  |  |  |  |  |  |  |  |  |  |  |  |  | | 8,9-DiHETrE | |  |  |  |  |  |
|  |  |  |  |  |  |  |  |  |  |  |  |  |  |  |  |  | 14,15-DiHETrE | |  |  |  |  |  |
|  |  |  |  |  |  |  |  |  |  |  |  |  |  |  |  |  | 12,20-DiHETE | |  |  |  |  |  |
|  |  |  |  |  |  |  |  |  |  |  |  |  |  |  |  |  | 5-HPETE | |  |  |  |  |  |
|  |  |  |  |  |  |  |  |  |  |  |  |  |  |  |  |  | 8(S)-HPETE | |  |  |  |  |  |
|  |  |  |  |  |  |  |  |  |  |  |  |  |  |  |  |  | 11(R)-HPETE | |  |  |  |  |  |
|  |  |  |  |  |  |  |  |  |  |  |  |  |  |  |  |  | 12(R)-HPETE | |  |  |  |  |  |
|  |  |  |  |  |  |  |  |  |  |  |  |  |  |  |  |  | 15(S)-HPETE | |  |  |  |  |  |
|  |  |  |  |  |  |  |  |  |  |  |  |  |  |  |  |  | 12(S)-HPETE | |  |  |  |  |  |
| **HORMONES** | 11beta-Hydroxy-3,20-dioxopregn-4-en-21-oic acid | 17a-Estradiol | | Methyltestosterone | | 6-Dehydrotestosterone glucuronide | 6-Dehydrotestosterone glucuronide | 6-Dehydrotestosterone glucuronide | | Methyltestosterone | | | 6-Dehydrotestosterone glucuronide | | 6-Dehydrotestosterone glucuronide | |  | | Methyltestosterone | 6-Dehydrotestosterone glucuronide | 6-Dehydrotestosterone glucuronide | |  |
|  | Estradiol-17beta 3-sulfate | Estradiol | | 6-Dehydrotestosterone glucuronide | | 11beta-Hydroxy-3,20-dioxopregn-4-en-21-oic acid | 11beta20-Dihydroxy-3-oxopregn-4-en-21-oic acid | 11beta20-Dihydroxy-3-oxopregn-4-en-21-oic acid | | 6-Dehydrotestosterone glucuronide | | | 5a-Dihydrotestosterone sulfate | | Methyltestosterone | |  |  | 6-Dehydrotestosterone glucuronide | Estradiol-17beta 3-sulfate |  | |  |
|  |  | Methyltestosterone | |  | | 21-Hydroxy-5b-pregnane-3,11,20-trione |  |  | | 11beta-Hydroxy-3,20-dioxopregn-4-en-21-oic acid | | | Androsterone sulfate | | 11beta-Hydroxy-3,20-dioxopregn-4-en-21-oic acid | |  |  |  |  |  |  |  |
|  |  |  | |  |  | 19-Hydroxydeoxycorticosterone |  |  |  |  | | |  | |  | |  |  |  |  |  |  |  |
|  |  |  |  |  |  | 21-Deoxycortisol |  |  |  |  |  |  |  |  |  |  |  |  |  |  |  |  |  |
|  |  |  |  |  |  | Corticosterone |  |  |  |  |  |  |  |  |  |  |  |  |  |  |  |  |  |
|  |  |  |  |  |  | Cortexolone |  |  |  |  |  |  |  |  |  |  |  |  |  |  |  |  |  |
| **PURINES, PYRIMIDINES** |  | 5-Aminoimidazole ribonucleotide | | 5-Methylthioadenosine | | 5-Methylthioadenosine | 5-Methylthioadenosine |  | | 5-Methylthioadenosine | | | 5-Methylthioadenosine | | 5-Methylthioadenosine | | 5-Methylthioadenosine | | 5-Methylthioadenosine | 5-Methylthioadenosine | 5-Methylthioadenosine | |  |
|  |  |  | | Cytidine 2,3-cyclic phosphate | | Cytosine | Cytidine 2,3-cyclic phosphate |  |  | Biotinyl-5-AMP | | | Cytidine 2,3-cyclic phosphate | | Cytidine 2,3-cyclic phosphate | | Cytidine 2,3-cyclic phosphate | | Cytidine 2,3-cyclic phosphate | Cytidine 2,3-cyclic phosphate | Cytidine 2,3-cyclic phosphate | |  |
|  |  |  |  | Cytidine monophosphate | | Deoxycytidine |  |  |  | 2-Deoxyinosine triphosphate | | | Cytidine monophosphate | | Deoxycytidine | |  | | 2-Deoxyinosine triphosphate | Deoxycytidine | Deoxycytidine | |  |
|  |  |  |  |  | |  |  |  |  |  | | | 2-Deoxyinosine triphosphate | | Cytosine | |  |  | Cytidine monophosphate | Cytosine | Cytosine | |  |
|  |  |  |  |  |  |  |  |  |  |  |  |  |  | |  | |  |  | Cytosine |  |  | |  |
| **LIPIDS** | 3-O-Sulfogalactosylceramide (d18:124:0) | 3-O-Sulfogalactosylceramide (d18:122:0) | | 3-O-Sulfogalactosylceramide (d18:122:0) | | 3-O-Sulfogalactosylceramide (d18:126:1(17Z)) | 3-O-Sulfogalactosylceramide (d18:124:0) | 3-O-Sulfogalactosylceramide (d18:122:0) | | 3-O-Sulfogalactosylceramide (d18:124:0) | | | 3-O-Sulfogalactosylceramide (d18:122:0) | | 3-O-Sulfogalactosylceramide (d18:122:0) | | 3-O-Sulfogalactosylceramide (d18:122:0) | | 3-O-Sulfogalactosylceramide (d18:124:0) | 3-O-Sulfogalactosylceramide (d18:122:0) | 3-O-Sulfogalactosylceramide (d18:122:0) | |  |
|  | 3-O-Sulfogalactosylceramide (d18:124:1(15Z)) | 3-O-Sulfogalactosylceramide (d18:124:0) | | 3-O-Sulfogalactosylceramide (d18:124:0) | | 3-O-Sulfogalactosylceramide (d18:122:0) | 3-O-Sulfogalactosylceramide (d18:120:0) | 3-O-Sulfogalactosylceramide (d18:122:0) | | 3-O-Sulfogalactosylceramide (d18:120:0) | | | 3-O-Sulfogalactosylceramide (d18:124:0) | | 3-O-Sulfogalactosylceramide (d18:124:0) | | 3-O-Sulfogalactosylceramide (d18:124:0) | | 3-O-Sulfogalactosylceramide (d18:124:1(15Z)) | 3-O-Sulfogalactosylceramide (d18:124:0) | 3-O-Sulfogalactosylceramide (d18:124:0) | |  |
|  | Oleamide | Oleamide | | Oleamide | | 3-O-Sulfogalactosylceramide (d18:120:0) | 3-O-Sulfogalactosylceramide (d18:122:0) | Oleamide | | Oleamide | | | Oleamide | | Oleamide | | Oleamide | | 3-O-Sulfogalactosylceramide (d18:122:0) |  | Oleamide | |  |
|  | Arachidonic acid |  | |  | | 3-O-Sulfogalactosylceramide (d18:124:0) | Oleamide |  | | Arachidonic acid | | | Arachidonic acid | | Arachidonic acid | |  | | 3-O-Sulfogalactosylceramide (d18:120:0) |  |  | |  |
|  |  |  |  |  |  | Oleamide | Arachidonic acid |  |  |  | | |  | |  | |  |  | Oleamide |  |  |  |  |
| **OTHER** | Taurine |  | | L-Urobilin | | L-Urobilin | cyclic 6-Hydroxymelatonin | Betaine | | Farnesyl pyrophosphate | | | Pentaporphyrin I | | cyclic 6-Hydroxymelatonin | | Norepinephrine sulfate | | Taurine | Norepinephrine sulfate | cyclic 6-Hydroxymelatonin | |  |
|  |  |  |  | Melanin | | N-Acetylserotonin sulfate | Betaine | Melatonin glucuronide | | Urothion | | | L-Urobilin | | Norepinephrine sulfate | | Taurine | | cyclic 6-Hydroxymelatonin | Taurine | Melatonin glucuronide | |  |
|  |  |  |  | Farnesyl pyrophosphate | | Melatonin glucuronide | L-Urobilin | Arachidonoyl Serinol | | Porphobilinogen | | | Melanin | | Farnesyl pyrophosphate | | 5-Hydroxyindoleacetic acid | | Norepinephrine sulfate |  | Porphobilinogen | |  |
|  |  |  |  | Betaine | | Porphobilinogen | Taurine |  | |  | | | Melatonin glucuronide | | Taurine | |  | | Melatonin glucuronide |  | L-Urobilin | |  |
|  |  |  |  |  | | 5-Hydroxyindoleacetic acid |  |  |  |  |  |  | Norepinephrine sulfate | |  | |  |  | L-Urobilin |  | L-Urobilinogen | |  |
|  |  |  |  |  |  |  |  |  |  |  |  |  | Taurine | |  |  |  |  |  |  |  | |  |
|  |  |  |  |  |  |  |  |  |  |  |  |  | Betaine | |  |  |  |  |  |  |  |  |  |

**Table S2.** Comparison of detected features in fresh perfusate samples (on-site SPME sampling) and in stored perfusate samples (in lab SPME sampling). Unique and multiple features with medium-to-high confidence matches annotated by HMDB were selected. The annotation was based on intensity profiles, retention time, mass defect, and isotope/adduct patterns of peaks. In high confidence match, non-zero xMSannotator multistage score, required adducts, N, O, P, S/C ratio check, hydrogen/carbon ratio check, abundance ratio checks for isotopes, multimers and multiply charged adducts are satisfied; in medium confidence match, pathway level correlation is satisfied.

|  | **1st h** | | **3rd h** | **5th h** | | **8th h** | **11th-12th h** | | **13th-14th h** | | **17th-19th h** |
| --- | --- | --- | --- | --- | --- | --- | --- | --- | --- | --- | --- |
|  | **FIBERS** | **BLADES** | **FIBERS** | **FIBERS** | **BLADES** | **FIBERS** | **FIBERS** | **BLADES** | **FIBERS** | **BLADES** | **BLADES** |
| **AMINO ACIDS** | L-Histidinol | Histidinal | N-Acetylhistamine | N-Acetylhistamine | NaNa-Dimethylhistamine | N-Acetylhistamine | N-Acetylhistamine | Na,Na-Dimethylhistamine | N-Acetylhistamine | Na,Na-Dimethylhistamine | Na,Na-Dimethylhistamine |
|  | N-Acetylhistamine | L-Histidine | L-Phenylalanine | Indoleacetyl glutamine | N-Acetylhistamine | Beta-Leucine | L-Histidinol | N-Acetylhistamine | L-Phenylalanine | N-Acetylhistamine | Histidinal |
|  | D-Leucine | L-Glutamine | L-Tyrosine | Quinaldic acid | 3-Methylhistamine | L-Cystine | L-Glutamine | L-Histidine | L-Tryptophan | Histidinal | L-Histidinol |
|  | N-acetyltryptophan | L-Asparagine | L-Tryptophan | Indoleacetyl glutamine | 1-Methylhistamine | L-Norleucine | L-Proline | Histidinal | Pyroglutamic acid | L-Histidinol | L-Histidine |
|  | Beta-Leucine | L-Threonine | L-Valine | L-Cystine | Histidinal | L-Tryptophan | N-acetyltryptophan | L-Histidinol | L-Phenylalanine | N-acetyltryptophan | N(6)-(Octanoyl)lysine |
|  | L-Norleucine | L-Proline | Kynurenic acid | D-Leucine | L-Histidinol | L-Homoserine | L-Glutamine | N-acetyltryptophan | Indoleacetyl glutamine | N-Acetylserotonin | L-Tryptophan |
|  | L-Tryptophan | L-Phenylalanine | Pyroglutamic acid | D-Tryptophan | N-Acetylvaline | L-Leucine | L-Tryptophan | L-Tryptophan | L-Valine | N-Decanoylglycine | Indoleacetyl glutamine |
|  | L-Leucine | Creatine | Indoleacetyl glutamine | L-Allothreonine | Creatinine | L-Glutamine | Kynurenic acid | Indoleacetyl glutamine | L-Methionine | Indoleacetyl glutamine | L-Tryptophan |
|  | L-Alloisoleucine | 5-Hydroxykynurenine | N-acetyltryptophan | Alanylglycine | L-Proline | L-Alloisoleucine | L-Leucine | N(6)-(Octanoyl)lysine | L-Glutamine | N(6)-(Octanoyl)lysine | Creatinine |
|  | L-Isoleucine | L-3-Hydroxykynurenine | Creatine | Beta-Leucine | Methylglutaric acid | L-Allothreonine | Pyroglutamic acid | L-Cystine | L-Arginine | Ornithine | Ornithine |
|  | L-Phenylalanine | Cycloserine | L-Methionine | D-Glutamine | Ketoleucine | Pyroglutamic acid | L-Isoleucine | L-Glutamine | L-Cystine | L-Proline | L-Proline |
|  | Indoleacetyl glutamine | L-Tryptophan | L-Glutamine | L-Norleucine | Pyroglutamic acid | L-Isoleucine | L-Carnitine | Creatinine | L-Histidine | N-Acetylvaline | Creatine |
|  | Tridecanoylglycine | Methylglutaric acid | Indoleacetyl glutamine | L-Tryptophan | Ornithine | L-Threonine | Valproylglycine | L-Arginine | L-Threonine | L-Asparagine | NeNe dimethyllysine |
|  | Tetracosanoylglycine | Hydroxykynurenine | Capryloylglycine | L-Homoserine | L-Fucose | L-Phenylalanine | N-Acryloylglycine | Ornithine | L-Tyrosine | L-Carnitine | N(6)-(Octanoyl)lysine |
|  | N-Undecanoylglycine | 2-Methylglutaric acid | Tridecanoylglycine | L-Leucine | 3-Methoxytyramine | Creatine | Capryloylglycine | L-Lysine | L-Glutamic acid | Creatinine | Farnesylcysteine |
|  | N-Nonanoylglycine | Pyroglutamic acid | Stearoylglycine | L-Glutamine | N-Acetyl-L-glutamate 5-semialdehyde | Indoleacetyl glutamine | Tridecanoylglycine | L-Asparagine | Indoleacetyl glutamine | Indoleacetaldehyde | N-Acetyl-L-glutamate 5-semialdehyde |
|  |  | Ketoleucine | Pristanoylglycine | L-Alloisoleucine | D-Serine | N-Undecanoylglycine | Stearoylglycine | L-Proline | N-Acryloylglycine | 23-Dimethyl-3-hydroxyglutaric acid | N6N6N6-Trimethyl-L-lysine |
|  |  | Dimethylglycine | N-Undecanoylglycine | Pyroglutamic acid | L-Tryptophan | Palmitoylglycine | N-Undecanoylglycine | Allysine | Tridecanoylglycine | Ketoleucine | L-Glutamic acid 5-phosphate |
|  |  | NaNa-Dimethylhistamine | N-Nonanoylglycine | L-Isoleucine | L-Tyrosine | Tridecanoylglycine | N-Nonanoylglycine | Methylglutaric acid | Tetracosanoylglycine | Ribitol | Quinaldic acid |
|  |  | Indoleacetyl glutamine | N-Acryloylglycine | L-Threonine | Indoleacetyl glutamine | Tetracosanoylglycine | N-Lauroylglycine | Urocanic acid | N-Undecanoylglycine | Urocanic acid | L-Glutamine |
|  |  | N-acetyltryptophan |  | L-Phenylalanine | N(6)-(Octanoyl)lysine | N-Nonanoylglycine | N-Lauroylglycine | Pyroglutamic acid | N-Nonanoylglycine | N-Acetyl-L-glutamate 5-semialdehyde | L-Cysteine |
|  |  | 8-Methoxykynurenate |  | Creatine | L-Cystine | N-Nonanoylglycine | N-Decanoylglycine | L-Phenylalanine | N-Lauroylglycine | D-Serine | L-Arginine |
|  |  | L-Cystine |  | Tridecanoylglycine | o-Tyrosine | N-Lauroylglycine | Palmitoylglycine | NeNe dimethyllysine | N-Decanoylglycine | N-Undecanoylglycine | L-Cystine |
|  |  | Quinaldic acid |  | Tetracosanoylglycine | Beta-Tyrosine | N-Lauroylglycine |  | N(6)-(Octanoyl)lysine | Palmitoylglycine | Valproylglycine | L-Lysine |
|  |  | N-Undecanoylglycine |  | N-Nonanoylglycine | L-Threo-3-Phenylserine | N-Decanoylglycine |  | D-Serine | N-Undecanoylglycine | N-Heptanoylglycine | L-Asparagine |
|  |  | N-Nonanoylglycine |  | N-Lauroylglycine | N-Undecanoylglycine | Valproylglycine |  | N6N6N6-Trimethyl-L-lysine |  | Valerylglycine | L-Threonine |
|  |  | N-Acryloylglycine |  | N-Decanoylglycine | N-Decanoylglycine | 5-L-Glutamylglycine |  | Citrulline |  | Capryloylglycine | L-Tyrosine |
|  |  | N-Decanoylglycine |  | Palmitoylglycine | Tridecanoylglycine | Alanylglycine |  | L-Valine |  | Isovalerylglycine | L-Glutamic acid |
|  |  |  |  | N-Acryloylglycine | N-Nonanoylglycine | N-Acryloylglycine |  | Quinaldic acid |  | 2-Methylbutyrylglycine | L-Carnitine |
|  |  |  |  | Tridecanoylglycine | Myristoylglycine | Capryloylglycine |  | Ureidosuccinic acid |  | Tridecanoylglycine | Kynurenic acid |
|  |  |  |  | N-Undecanoylglycine | N-Acryloylglycine | Tridecanoylglycine |  | L-Methionine |  | N-Lauroylglycine | 2-Methylglutaric acid |
|  |  |  |  | N-Nonanoylglycine | Valerylglycine | N-Decanoylglycine |  | L-Cystine |  | Myristoylglycine | N-Undecanoylglycine |
|  |  |  |  | N-Undecanoylglycine | Isovalerylglycine |  |  | L-Threonine |  |  | N-Lauroylglycine |
|  |  |  |  | Valproylglycine | N-Nonanoylglycine |  |  | L-Tyrosine |  |  | N-Decanoylglycine |
|  |  |  |  | 5-L-Glutamylglycine | N-Undecanoylglycine |  |  | L-Glutamic acid |  |  | N-Undecanoylglycine |
|  |  |  |  |  | N-Lauroylglycine |  |  | Creatine |  |  | N-Nonanoylglycine |
|  |  |  |  |  | N-Decanoylglycine |  |  | 5-L-Glutamylglycine |  |  | N-Decanoylglycine |
|  |  |  |  |  |  |  |  | N-Acryloylglycine |  |  | Tridecanoylglycine |
|  |  |  |  |  |  |  |  | Tiglylglycine |  |  | Stearoylglycine |
|  |  |  |  |  |  |  |  | N-Butyrylglycine |  |  | Myristoylglycine |
|  |  |  |  |  |  |  |  | Isobutyrylglycine |  |  | Palmitoylglycine |
|  |  |  |  |  |  |  |  | N-Lauroylglycine |  |  | Valproylglycine |
|  |  |  |  |  |  |  |  | N-Decanoylglycine |  |  |  |
|  |  |  |  |  |  |  |  | N-Undecanoylglycine |  |  |  |
|  |  |  |  |  |  |  |  | N-Lauroylglycine |  |  |  |
|  |  |  |  |  |  |  |  | Palmitoylglycine |  |  |  |
|  |  |  |  |  |  |  |  | Tridecanoylglycine |  |  |  |
|  |  |  |  |  |  |  |  | Stearoylglycine |  |  |  |
| **PEPTIDES** |  | Valyl-Hydroxyproline | Arginyl-Phenylalanine | Phenylalanyl-Arginine | Valyl-Aspartate | Phenylalanyl-Arginine | Phenylalanyl-Arginine | Tryptophyl-Arginine | Phenylalanyl-Arginine | Arginyl-Arginine | Arginyl-Arginine |
|  |  | Prolyl-Asparagine |  | Aspartyl-Alanine | Threoninyl-Hydroxyproline | Aspartyl-Alanine | Arginyl-Phenylalanine | Phenylalanyl-Arginine | Arginyl-Phenylalanine |  | gamma-Glutamyl-S-methylcysteinyl-beta-alanine |
|  |  | Phenylalanyl-Arginine |  | Arginyl-Phenylalanine | Hydroxyprolyl-Threonine | Arginyl-Phenylalanine |  | Histidinyl-Alanine |  |  |  |
|  |  | Hydroxyprolyl-Valine |  | Alanyl-Glycine | Aspartyl-Valine | Alanyl-Glycine |  | Aspartyl-Alanine |  |  |  |
|  |  | Aspartyl-Phenylalanine |  | Alanyl-Aspartate | Histidinyl-Cysteine | Alanyl-Aspartate |  | Arginyl-Tryptophan |  |  |  |
|  |  | Asparaginyl-Proline |  | Arginyl-Arginine | Cysteinyl-Histidine |  |  | Arginyl-Phenylalanine |  |  |  |
|  |  | Arginyl-Phenylalanine |  |  | Arginyl-Arginine |  |  | Alanyl-Histidine |  |  |  |
|  |  | Arginyl-Arginine |  |  |  |  |  | Alanyl-Aspartate |  |  |  |
| **ACYLCARNITINES** | Hydroxypropionylcarnitine | Propionylcarnitine | L-Acetylcarnitine | 6-Keto-decanoylcarnitine | L-Carnitine | 6-Keto-decanoylcarnitine | Arachidonyl carnitine | L-Acetylcarnitine | 6-Keto-decanoylcarnitine | Propionylcarnitine | 3-Dehydrocarnitine |
|  | Decanoylcarnitine | L-Acetylcarnitine | L-Carnitine | L-Acetylcarnitine | L-Acetylcarnitine | L-Acetylcarnitine | Malonylcarnitine | Propionylcarnitine | L-Acetylcarnitine | L-Acetylcarnitine | Propionylcarnitine |
|  |  | Malonylcarnitine | Malonylcarnitine |  | 3-Dehydrocarnitine |  | 3-Hydroxy-9-hexadecenoylcarnitine | Tiglylcarnitine |  | 3-Dehydroxycarnitine | L-Acetylcarnitine |
|  |  |  | 6-Keto-decanoylcarnitine |  | 3-Dehydroxycarnitine |  |  | 3-Dehydroxycarnitine |  | Tiglylcarnitine | Malonylcarnitine |
|  |  |  |  |  | Tiglylcarnitine |  |  | Hydroxypropionylcarnitine |  | Hydroxypropionylcarnitine | 2-Octenoylcarnitine |
|  |  |  |  |  | Propionylcarnitine |  |  | L-Carnitine |  | 3-Dehydrocarnitine | Heptanoylcarnitine |
|  |  |  |  |  | Hydroxypropionylcarnitine |  |  |  |  |  | Hydroxypropionylcarnitine |
|  |  |  |  |  |  |  |  |  |  |  | Tiglylcarnitine |
|  |  |  |  |  |  |  |  |  |  |  | Dodecanoylcarnitine |
|  |  |  |  |  |  |  |  |  |  |  | 3-Dehydroxycarnitine |
| **AUTACOIDS** |  |  | Resolvin D1 | Resolvin D1 |  | Resolvin D1 | Resolvin D5 | Resolvin D1 |  | Resolvin D1 | Resolvin D1 |
|  |  |  | Resolvin D2 | Resolvin D2 |  | Resolvin D2 | Neuroprotectin D1 | Resolvin D2 |  | Resolvin D2 | Resolvin D2 |
| **PROSTAGLANDINS** | PGF2a ethanolamide | 6-Keto-prostaglandin F1a | 7-hydroxy-D4-neuroprostane | 7-hydroxy-D4-neuroprostane | 15-Deoxy-d-12,14-PGJ2 | 7-hydroxy-D4-neuroprostane | Prostaglandin G1 | 7-hydroxy-D4-neuroprostane | 13,14-Dihydro PGF-1a | 7-hydroxy-D4-neuroprostane | 7-hydroxy-D4-neuroprostane |
|  |  | 15-Deoxy-d-12,14-PGJ2 | 4-hydroxy-D4-neuroprostane | 4-hydroxy-D4-neuroprostane |  | 4-hydroxy-D4-neuroprostane | 20-Hydroxy-PGF2a | 4-hydroxy-D4-neuroprostane |  | 4-hydroxy-D4-neuroprostane | 4-hydroxy-D4-neuroprostane |
|  |  | Prostaglandin G1 | 20-hydroxy-E4-neuroprostane | 20-hydroxy-E4-neuroprostane |  | 20-hydroxy-E4-neuroprostane |  | 20-hydroxy-E4-neuroprostane |  | 20-hydroxy-E4-neuroprostane | 20-hydroxy-E4-neuroprostane |
|  |  | 20-Hydroxy-PGF2a | 17-hydroxy-E4-neuroprostane | 17-hydroxy-E4-neuroprostane |  | 17-hydroxy-E4-neuroprostane |  | 17-hydroxy-E4-neuroprostane |  | 17-hydroxy-E4-neuroprostane | 17-hydroxy-E4-neuroprostane |
|  |  |  | 14-hydroxy-E4-neuroprostane | 14-hydroxy-E4-neuroprostane |  | 14-hydroxy-E4-neuroprostane |  | 14-hydroxy-E4-neuroprostane |  | 14-hydroxy-E4-neuroprostane | 14-hydroxy-E4-neuroprostane |
|  |  |  |  | 15-Deoxy-d-12,14-PGJ2 |  | 15-Deoxy-d-12,14-PGJ2 |  | 6-Keto-prostaglandin F1a |  | 20-hydroxy-E4-neuroprostane | Prostaglandin D2 |
|  |  |  |  |  |  |  |  | PGF2a ethanolamide |  | 15-Deoxy-d-12,14-PGJ2 | Prostaglandin H2 |
|  |  |  |  |  |  |  |  | 20-Hydroxy-PGF2a |  |  | Prostaglandin I2 |
|  |  |  |  |  |  |  |  |  |  |  | Prostaglandin E2 |
|  |  |  |  |  |  |  |  |  |  |  | PGF2a ethanolamide |
|  |  |  |  |  |  |  |  |  |  |  | 13,14-Dihydro-15-keto-PGD2 |
|  |  |  |  |  |  |  |  |  |  |  | 11b-PGE2 |
|  |  |  |  |  |  |  |  |  |  |  | 8-iso-15-keto-PGF2a |
|  |  |  |  |  |  |  |  |  |  |  | 15-Keto-prostaglandin F2a |
|  |  |  |  |  |  |  |  |  |  |  | 8-iso-PGF3a |
|  |  |  |  |  |  |  |  |  |  |  | Prostaglandin F3a |
|  |  |  |  |  |  |  |  |  |  |  | 56-Dihydroxyprostaglandin F1a |
|  |  |  |  |  |  |  |  |  |  |  | (5Z)-(15S)-11alpha-Hydroxy-9,15-dioxoprostanoate |
| **THROMBOXANES** |  | Thromboxane B2 |  |  |  |  | Thromboxane B2 | Thromboxane B2 |  |  | Thromboxane A2 |
| **LEUKOTRIENES** | Leukotriene C5 | 10,11-dihydro-20-dihydroxy-LTB4 | Leukotriene E4 | Omega-Carboxy-trinor-leukotriene B4 | 6,7-dihydro-12-epi-LTB4 | Omega-Carboxy-trinor-leukotriene B4 | 12-Oxo-20-trihydroxy-leukotriene B4 | 10,11-dihydro-20-dihydroxy-LTB4 | Leukotriene E4 |  | 10,11-dihydro-20-trihydroxy-leukotriene B4 |
|  | Leukotriene E4 |  | Omega-Carboxy-trinor-leukotriene B4 | Leukotriene C5 | 10,11-dihydro-leukotriene B4 | Leukotriene C5 | 10,11-dihydro-20-dihydroxy-LTB4 | 10,11-dihydro-20-trihydroxy-leukotriene B4 |  |  | Leukotriene B4 ethanolamide |
|  | Omega-Carboxy-trinor-leukotriene B4 |  | Leukotriene C5 | Leukotriene E4 | 12-Keto-tetrahydro-leukotriene B4 | Leukotriene E4 | Leukotriene E4 |  |  |  | 20-Hydroxy-leukotriene B4 |
|  | 12-Oxo-20-trihydroxy-leukotriene B4 |  |  |  |  |  | 10,11-dihydro-20-trihydroxy-leukotriene B4 |  |  |  |  |
|  |  |  |  |  |  |  |  | 9-HODE |  |  |  |
|  |  |  |  |  |  |  |  | 15-Deoxy-d-12,14-PGJ2 |  |  |  |
|  |  |  |  |  |  |  |  | Prostaglandin G1 |  |  |  |
| **LIPOXINS** |  |  |  |  |  |  |  |  |  |  | 15-Epi-lipoxin A4 |
|  |  |  |  |  |  |  |  |  |  |  | 13,14-Dihydro-15-oxo-lipoxin A4 |
| **OTHER OXYLIPINS** |  | 9,10,13-TriHOME |  | 9,10-DiHODE | 5,6-DHET | 9,10-DiHODE |  | 9,10,13-TriHOME | 12(13)Ep-9-KODE | Tetranor 12-HETE | 9-HODE |
|  |  | 9,12,13-TriHOME |  | 15,16-DiHODE | 11,12-DiHETrE | 15,16-DiHODE |  | 9,12,13-TriHOME | 9,10,13-TriHOME | 9,10,13-TriHOME | 9,10-DiHODE |
|  |  |  |  | 12,13-DiHODE | 8,9-DiHETrE | 12,13-DiHODE |  | 12,13-EpOME | 9,12,13-TriHOME | 9,12,13-TriHOME | 15,16-DiHODE |
|  |  |  |  | 9(S)-HPODE | 14,15-DiHETrE | 9(S)-HPODE |  | Tetranor 12-HETE |  |  | 12,13-DiHODE |
|  |  |  |  | 9,10,13-TriHOME | 9,10,13-TriHOME | 9,10,13-TriHOME |  | 9(S)-HPODE |  |  | 9(S)-HPODE |
|  |  |  |  | 9,12,13-TriHOME | 9,12,13-TriHOME | 9,12,13-TriHOME |  |  |  |  | 9,10,13-TriHOME |
|  |  |  |  |  |  |  |  |  |  |  | 9,12,13-TriHOME |
|  |  |  |  |  |  |  |  |  |  |  | 12,13-EpOME |
| **HORMONES** | 17a-Estradiol | 19-Oxo-deoxycorticosterone | 17a-Estradiol | 11beta-Hydroxy-3,20-dioxopregn-4-en-21-oic acid | Methyltestosterone | 17a-Estradiol | 17a-Estradiol | 11beta-Hydroxy-3,20-dioxopregn-4-en-21-oic acid | 5b-Dihydrotestosterone | Cortisone | 21-Deoxycortisol |
|  | Estradiol | 19-Hydroxydeoxycorticosterone | Estradiol | Estradiol | 19-Oxo-deoxycorticosterone | Estradiol | Estradiol | 19-Oxo-deoxycorticosterone | 4-Androstenediol | 18-Oxocortisol | 6-Dehydrotestosterone glucuronide |
|  |  | 2-Hydroxy-3-methoxyestrone | 11beta-Hydroxy-3,20-dioxopregn-4-en-21-oic acid | 6-Dehydrotestosterone glucuronide | 19-Hydroxydeoxycorticosterone | 6-Dehydrotestosterone glucuronide | 6-Dehydrotestosterone glucuronide | 19-Hydroxydeoxycorticosterone | 5-Androstenediol | Aldosterone | 19-Oxo-deoxycorticosterone |
|  |  | Adrenosterone | 6-Dehydrotestosterone glucuronide | 17a-Estradiol | 11beta20-Dihydroxy-3-oxopregn-4-en-21-oic acid | Methyltestosterone |  | 11beta20-Dihydroxy-3-oxopregn-4-en-21-oic acid | Dihydrotestosterone | 11beta-Hydroxy-3,20-dioxopregn-4-en-21-oic acid | 19-Hydroxydeoxycorticosterone |
|  |  | 19-Oxoandrost-4-ene-3,17-dione |  |  | 21-Hydroxy-5b-pregnane-3,11,20-trione | 11beta-Hydroxy-3,20-dioxopregn-4-en-21-oic acid |  | Adrenosterone | Epiandrosterone | 11beta20-Dihydroxy-3-oxopregn-4-en-21-oic acid | 11beta-Hydroxy-320-dioxopregn-4-en-21-oic acid |
|  |  | 21-Hydroxy-5b-pregnane-3,11,20-trione |  |  | 21-Deoxycortisol |  |  | 19-Oxoandrost-4-ene-3,17-dione | Androsterone |  | 11beta20-Dihydroxy-3-oxopregn-4-en-21-oic acid |
|  |  | 6b-Hydroxymethandienone |  |  | 11-Dehydrocorticosterone |  |  | 21-Hydroxy-5b-pregnane-3,11,20-trione | 6-Dehydrotestosterone glucuronide |  | 21-Hydroxy-5b-pregnane-31120-trione |
|  |  | 21-Deoxycortisol |  |  | 18-Oxocortisol |  |  | 21-Deoxycortisol | 11beta20-Dihydroxy-3-oxopregn-4-en-21-oic acid |  | 21-Deoxycortisol |
|  |  | 11-Dehydrocorticosterone |  |  | Cortexolone |  |  | 11-Dehydrocorticosterone |  |  | 11-Dehydrocorticosterone |
|  |  | Corticosterone |  |  | 3b16a-Dihydroxyandrostenone sulfate |  |  | Corticosterone |  |  | Corticosterone |
|  |  | Cortexolone |  |  | Corticosterone |  |  | 3b16a-Dihydroxyandrostenone sulfate |  |  | Androstanedione |
|  |  | 2-Methoxyestrone |  |  |  |  |  | Cortexolone |  |  | Epitestosterone |
|  |  | 3b16a-Dihydroxyandrostenone sulfate |  |  |  |  |  | 2-Methoxyestrone |  |  | 18-Oxocortisol |
|  |  |  |  |  |  |  |  | 6-Dehydrotestosterone glucuronide |  |  | Testosterone |
|  |  |  |  |  |  |  |  |  |  |  | Dehydroepiandrosterone |
|  |  |  |  |  |  |  |  |  |  |  | Cortexolone |
|  |  |  |  |  |  |  |  |  |  |  | 3b16a-Dihydroxyandrostenone sulfate |
|  |  |  |  |  |  |  |  |  |  |  | 21-Hydroxy-5b-pregnane-31120-trione |
|  |  |  |  |  |  |  |  |  |  |  | 19-Hydroxydeoxycorticosterone |
| **PURINES, PYRIMIDINES** | 6-Thioinosinic acid | Cytosine | 5-Aminoimidazole ribonucleotide | Cytidine 2,3-cyclic phosphate | Deoxyadenosine monophosphate | Cytidine 2,3-cyclic phosphate | 5-Methylthioadenosine | 5-Methylthioadenosine | Cytidine monophosphate | dUMP | dUMP |
|  | Cytidine 2,3-cyclic phosphate | Cytidine monophosphate | Allopurinol riboside | 5-Methylthioadenosine | Biotinyl-5-AMP | 5-Methylthioadenosine |  | Deoxyadenosine monophosphate | 5-Methylthioadenosine | 3-Methylcytosine | 3-Methylcytosine |
|  | 2-Deoxyinosine triphosphate | 3-Methylcytosine |  |  | dUMP |  |  | Cytidine 2,3-cyclic phosphate |  | 2-O-Methylcytosine | 2-O-Methylcytosine |
|  | 5-Methylthioadenosine | 5-Methylcytosine |  |  | 3-Methylcytosine |  |  | Cytidine monophosphate |  | 5-Methylcytosine | 8-Hydroxypurine |
|  |  | Dihydrouracil |  |  | Cytosine |  |  | Cysteine-S-sulfate |  | Thymine | Thiocysteine |
|  |  | 2-O-Methylcytosine |  |  |  |  |  | Cytosine |  | Dihydrouracil | 5-Methylthioadenosine |
|  |  |  |  |  |  |  |  | Dihydrouracil |  | 8-Hydroxypurine | Cytosine |
|  |  |  |  |  |  |  |  | Deoxycytidine |  |  | Deoxycytidine |
|  |  |  |  |  |  |  |  | 8-Hydroxypurine |  |  | 13-Dimethyluracil |
|  |  |  |  |  |  |  |  |  |  |  | N-Acetyl-S-(N-methylcarbamoyl)cysteine |
|  |  |  |  |  |  |  |  |  |  |  | 24-Diamino-6-hydroxypyrimidine |
|  |  |  |  |  |  |  |  |  |  |  | Dihydrothymine |
|  |  |  |  |  |  |  |  |  |  |  | 6-Dimethylaminopurine |
|  |  |  |  |  |  |  |  |  |  |  | 5-Thymidylic acid |
| **LIPIDS** | 3-O-Sulfogalactosylceramide (d18:122:0) | Oleamide | 3-O-Sulfogalactosylceramide (d18:124:0) | 3-O-Sulfogalactosylceramide (d18:120:0) |  | 3-O-Sulfogalactosylceramide (d18:122:0) | 3-O-Sulfogalactosylceramide (d18:122:0) | 3-O-Sulfogalactosylceramide (d18:124:0) | 3-O-Sulfogalactosylceramide (d18:122:0) |  | 3-O-Sulfogalactosylceramide (d18:124:1(15Z)) |
|  | 3-O-Sulfogalactosylceramide (d18:124:0) |  | 3-O-Sulfogalactosylceramide (d18:126:1(17Z)) | 3-O-Sulfogalactosylceramide (d18:124:0) |  | 3-O-Sulfogalactosylceramide (d18:120:0) | 3-O-Sulfogalactosylceramide (d18:124:0) | 3-O-Sulfogalactosylceramide (d18:124:1(15Z)) | 3-O-Sulfogalactosylceramide (d18:120:0) |  | 3-O-Sulfogalactosylceramide (d18:122:0) |
|  | Oleamide |  | 3-O-Sulfogalactosylceramide (d18:122:0) |  |  | 3-O-Sulfogalactosylceramide (d18:124:0) |  | 3-O-Sulfogalactosylceramide (d18:120:0) | Arachidonic acid |  | 3-O-Sulfogalactosylceramide (d18:120:0) |
|  |  |  | Arachidonic acid |  |  |  |  | 3-O-Sulfogalactosylceramide (d18:118:0) |  |  | 3-O-Sulfogalactosylceramide (d18:118:0) |
|  |  |  |  |  |  |  |  | 3-O-Sulfogalactosylceramide (d18:112:0) |  |  | 3-O-Sulfogalactosylceramide (d18:124:0) |
|  |  |  |  |  |  |  |  | Oleamide |  |  | 3-O-Sulfogalactosylceramide (d18:112:0) |
|  |  |  |  |  |  |  |  |  |  |  | Arachidonic acid |
|  |  |  |  |  |  |  |  |  |  |  | Oleamide |
| **OTHER** | Melatonin glucuronide | Urothion | Mevalonic acid-5P |  | D-Fucose | Taurine | Norepinephrine sulfate | Allantoin | NNAL-N-glucuronide | Histamine Phosphate | Lipoyl-AMP |
|  | cyclic 6-Hydroxymelatonin | Fucose 1-phosphate | 2-Aminomuconic acid |  | Fucose 1-phosphate |  | Farnesyl pyrophosphate | Porphobilinogen | Taurine | Acetylcholine | Biotinyl-5-AMP |
|  | Farnesyl pyrophosphate | Allantoin | Melatonin glucuronide |  | Allantoin |  | Melatonin glucuronide | dUMP |  | Fucose 1-phosphate | Porphobilinogen |
|  | Taurine | Serotonin | cyclic 6-Hydroxymelatonin |  | Porphobilinogen |  | L-Urobilin | Neuromedin N (1-4) |  | Allantoin | Bilirubin |
|  |  | cyclic 6-Hydroxymelatonin | Betaine |  |  |  |  | Fucose 1-phosphate |  | Taurine | Melatonin glucuronide |
|  |  | Melatonin glucuronide | Taurine |  |  |  |  | Taurine |  | Porphobilinogen | Allantoic acid |
|  |  | Melanin | L-Urobilin |  |  |  |  | Melanin |  | 3-O-Methyl-a-methyldopamine | FAPy-adenine |
|  |  | Ribitol | Melanin |  |  |  |  | Norepinephrine sulfate |  |  | L-Urobilin |
|  |  | L-Fucose |  |  |  |  |  | L-Urobilin |  |  | Melanin |
|  |  | Histamine Phosphate |  |  |  |  |  | Bilirubin |  |  | DOPA sulfate |
|  |  |  |  |  |  |  |  | Carnosine |  |  | Taurine |
|  |  |  |  |  |  |  |  | 4E15Z-Bilirubin IXa |  |  | Protoporphyrinogen IX |
|  |  |  |  |  |  |  |  |  |  |  | Phosphohydroxypyruvic acid |
|  |  |  |  |  |  |  |  | cyclic 6-Hydroxymelatonin |  |  | 4E,15Z-Bilirubin IXa |
|  |  |  |  |  |  |  |  | 3-O-Methyl-a-methyldopamine |  |  | Urocanic acid |
